# A comprehensive strategy for the development of a multi-epitope vaccine targeting *Treponema pallidum*, utilizing heat shock proteins, encompassing the entire process from vaccine design to *in vitro* evaluation of immunogenicity

**DOI:** 10.3389/fmicb.2025.1551437

**Published:** 2025-03-19

**Authors:** Jing Jiang, Linglan Xu, Xuefeng Wang, Ming Wang, Youde Cao, Ranhui Li, Kang Zheng, Xian Wu

**Affiliations:** ^1^The First Affiliated Hospital of Hunan Traditional Chinese Medical College (Hunan Province Directly Affiliated TCM Hospital), Zhuzhou, China; ^2^Department of Clinical Laboratory, Affiliated Hengyang Hospital of Hunan Normal University & Hengyang Central Hospital, Hengyang, China; ^3^Hunan Provincial Key Laboratory for Special Pathogens Prevention and Control, Institute of Pathogenic Biology, Hengyang Medical School, University of South China, Hengyang, China; ^4^Hunan Traditional Chinese Medical College, Zhuzhou, Hunan, China; ^5^Department of Clinical Laboratory, Hunan Provincial People’s Hospital (The First-Affiliated Hospital of Hunan Normal University), Changsha, Hunan, China

**Keywords:** *Treponema pallidum*, heat shock protein, reverse vaccinology, immunoinformatic, subtractive proteomics, immune stimulation, vaccine

## Abstract

**Background:**

*Treponema pallidum*, the causative spirochete of syphilis, is primarily transmitted through sexual contact and has emerged as a significant global health concern. To address this issue, enhancing diagnostic capabilities, strengthening public health interventions, and developing a safe and effective vaccine are critical strategies.

**Objective:**

This study employed an immunoinformatics approach to design a vaccine with high immunogenic potential, targeting the heat shock proteins of *T. pallidum*.

**Methods:**

Based on heat shock proteins of *T. pallidum*, we predicted B-cell, CTL, and HTL epitopes and all the selected epitopes were linked to construct a multi-epitope vaccine. Antigenicity, toxicity, and allergenicity of epitopes were checked by VaxiJen 2.0, AllerTOP v2.0, and ToxinPred servers. After constructing the multi-epitope vaccine, we subsequently predicted its secondary and tertiary protein structures. After refining and validating the modeled structure, we utilized advanced computational approaches, including molecular docking and dynamic simulations, to evaluate the binding affinity, compatibility, and stability of the vaccine-adjuvant complexes. Eventually, *in silico* cloning was conducted to optimize protein expression and production.

**Results:**

The multi-epitope subunit vaccine we developed was constructed by seven cytotoxic T lymphocyte epitopes, five helper T lymphocyte epitopes, four B cell epitopes, and adjuvant β-defensin. An adjuvant was used to enhance immune responses, all of which were linked to one another using GPGPG, AAY, and KK linkers, respectively. The population coverage of the designed vaccine was 94.41% worldwide. Molecular docking and MD simulations indicated strong binding interactions with TLR1/2, TLR-2 and TLR-4 in a stable vaccine-receptor complex. The final designed vaccine, composed of 502 amino acids, theoretically exhibits high antigenicity and immunity, capable of inducing both humoral and cellular immune responses.

**Conclusion:**

The vaccine developed in this study theoretically represents a safe and potent multi-epitope prophylactic strategy against *T. pallidum*, subject to further experimental validation to ascertain its actual protective efficacy.

## 1 Introduction

*Treponema* species comprise anaerobic or microaerophilic spirochetes that establish complex associations with their host organisms. These bacteria are classified within the family *Spirochaetaceae* (Stamm and Bergen, 2000). Syphilis, a well-documented sexually transmitted infection, is caused by the spirochetal bacterium *Treponema pallidum* subspecies *pallidum* (TPA). Although the origins of the syphilis pandemic remain enigmatic, historical evidence suggests that the earliest recorded cases of this disease appeared in Europe in the late 15th century (De Melo et al., 2010; Harper et al., 2011). Initially, syphilis and the three nonvenereal *treponematoses*—yaws, bejel, and pinta—were mistakenly attributed to a single causative agent, despite the distinct clinical manifestations associated with each condition. Notably, pinta is caused by a different species of spirochete bacteria, *Treponema carateum*. These diseases are transmitted by obligate human pathogens, which are well-knowne for their invasive properties and their proficiency in evading the host immune system (Radolf et al., 2016; Thomas et al., 1988; LaFond and Lukehart, 2006; Giacani and Lukehart, 2014). In 1998, a pivotal advancement in our comprehension of *Treponema pallidum* was achieved with the publication of the first complete genome sequence of the Nichols strain (Fraser et al., 1998). Recent advancements have facilitated the complete sequencing of multiple strains of *Treponema pallidum* responsible for syphilis, including SS14 (Matějková et al., 2008), Chicago (Giacani et al., 2010), and Amoy (Tong et al., 2017). These developments have significantly enhanced our genomic understanding of this pathogen.

In the aftermath of the AIDS epidemic, syphilis is considered the second most lethal sexually transmitted infection. This condition has the potential to cause systemic diseases in humans by indiscriminately damaging multiple organ systems (Ramos et al., 2007). In 1999, the World Health Organization (WHO) estimated that 12 million new cases of syphilis were identified, with over 90% occurring in less developed countries (Schmid et al., 2007). Unfortunately, the prevalence of syphilis has continued to rise steadily over the past decade (Zheng et al., 2022). According to World Health Organization (WHO) statistics, in 2016, the global prevalence of syphilis was estimated at 19.9 million individuals, with an alarming annual incidence of 6.3 million new cases (Rowley et al., 2019). Syphilis is acknowledged as the second most significant cause of stillbirths, surpassed only by malaria (Lawn et al., 2016). *T. pallidum* could penetrates the placental barrier through the paracellular route and transcytosis, leading to the stillbirths (Li et al., 2023). A considerable proportion of reported cases involve congenital syphilis, which has emerged as the leading cause of both miscarriages and neonatal deaths (Eppes et al., 2022; Qin et al., 2014).

Owing to the characteristic of motility, slow replication, exogenous polysaccharides and outer membrane, *T. pallidum* escape the host immune response (Tang et al., 2023). Despite the straightforward diagnosis and cost-effective antibiotic treatment of syphilis, coupled with the lack of an identified animal reservoir, the reemergence of this venereal disease among human populations represents a significant global health challenge.

Syphilis infection progresses through three distinct stages (Peeling et al., 2023). The initial phase, termed primary syphilis, generally appears approximately 2–3 weeks following exposure to the causative pathogen, characterized by the development of an ulcerative lesion known as a chancre. In the absence of treatment, this primary lesion typically resolves spontaneously within 3–6 weeks (Peeling et al., 2023; Hook, 2017). The subsequent stage, secondary syphilis, occurs as a result of systemic bacterial dissemination, and is associated with symptoms such as fatigue, headache, fever, generalized lymphadenopathy, and maculopapular rashes, which may manifest in localized or widespread areas of the body (Bhugra and Maiti, 2020). If left untreated, these secondary lesions can persist for several months. Subsequently, the condition enters a latent phase characterized by an absence of clinical symptoms (Trawinski, 2021). After several years or even decades, 15–40% of individuals who have not received treatment during this latent period may develop tertiary syphilis. This advanced stage is associated with severe complications affecting the cardiovascular, neurological, skeletal, and visceral systems, which can ultimately result in mortality (Peeling et al., 2023; Jankowska et al., 2022).

*Treponema pallidum* has consistently demonstrated susceptibility to penicillin, which has been the primary treatment for syphilis for over 70 years. Penicillin administration effectively halts the progression of syphilis to its secondary and tertiary stages and eradicates the infection (World Health Organization, 2016). In response to increasing challenges in treatment, the use of the oral antibiotic azithromycin has become more prevalent (Fica et al., 2019). Nevertheless, resistance to azithromycin and other macrolides is emerging, posing a significant challenge in the management of *T. pallidum* infections (Katz and Klausner, 2008). In 1964 and 1976, instances of erythromycin therapy failure in treating syphilis among pregnant women were recorded (Khan et al., 2022). Despite undergoing treatment, individuals remain vulnerable to reinfection, as previous exposure does not generate a strong immunological response. Therefore, there is an urgent need for the development of a syphilis vaccine to enhance existing strategies for controlling and preventing the transmission of this infectious disease.

Currently, there are no approved vaccines for preventing syphilis in humans. The development of a syphilis vaccine remains in the experimental stage. In the early 20th century, scientists tried to use inactivated or attenuated *T. pallidum* as a vaccine, but were unsuccessful. In the mid-20th century, with the development of molecular biology, researchers began to focus on the specific antigens of *T. pallidum* (such as TprK protein, Gpd protein, etc.), but these studies are still in the experimental stage (Li et al., 2023; Ávila-Nieto et al., 2023). Nucleic acid vaccine against *T. pallidum* including pcDNA/Gpd-IL-2 (Zhao et al., 2013; Zhang et al., 2018), plasmid DNA encoding flagellin pcDNA3/flab3 (Zheng et al., 2018), among others, have been developed in animal experimental stage. Currently, there is no licensed mRNA vaccine for this pathogen, and the development of traditional vaccines continues to face significant challenges.

Traditional vaccine production methods involve cultivating and isolating target microorganisms, subsequently characterizing them to identify and purify components capable of eliciting an immune response (Stevenson et al., 2018). While these techniques have historically been important, they are now recognized as costly and relatively inefficient. In contrast, the emerging discipline of immunoinformatics utilizes computational strategies to identify immunodominant epitopes within pathogen genomes, which are then used to develop innovative vaccine designs (Gomes et al., 2023). By integrating the methodologies of reverse vaccinology (RV) with immunoinformatics, researchers are pioneering novel pathways for identifying vaccine targets, which may accelerate the development process while reducing costs (Hegde et al., 2018). RV facilitates the identification of optimal candidate antigens, thus allowing for the formulation of vaccines that were previously unattainable (Srivastava and Jain, 2023). The designation “reverse” highlights the focus on expressed DNA sequences rather than the traditional approach of utilizing proteins isolated from organisms (Monterrubio-López and Delgadillo-Gutiérrez, 2021).

Prior studies have highlighted the use of *Treponema pallidum* outer membrane proteins (OMPs) as potential immunogens for inducing anti-*T. pallidum* immune responses and elucidating the bacterium’s evasion strategies (Ávila-Nieto et al., 2023). Expanding on this foundational work, Khan et al. (2022) employed RV techniques to develop a subtractive proteomics-based vaccine focused on *T. pallidum* OMPs. Moreover, multi-epitope vaccines based on the whole genome of *T. pallidum* has also been developed in recent years (Gomes et al., 2022). In the present study, we concentrated on the heat-shock protein (HSP) of *Treponema pallidum*. Heat-shock proteins are known to modulate physiological functions and virulence by interacting with diverse cellular signaling pathway regulators across a broad spectrum of organisms (Bolhassani and Agi, 2019). They play a crucial role in the cross-presentation of HSP-complexed peptides and are instrumental in the activation of dendritic cells (DC) (Zachova et al., 2016; Yin et al., 2024). Previous research has identified the presence of HSPs in the serum of patients with syphilis, suggesting an association with *T. pallidum* infection (Tong et al., 2021). However, research on heat-shock proteins (HSPs) in the context of syphilis remains limited, and the exact mechanisms of infection are not yet fully understood. The findings discussed indicate that HSPs hold promise as potential vaccine candidates, as evidenced by several HSP-based vaccines that have shown positive immunological responses against a range of microorganisms (Danladi and Sabir, 2021).

A multi-epitope vaccine was developed, incorporating a variety of antigenic epitopes and distinct linkers specifically designed for immunization purposes. Six heat shock proteins were selected for analysis from the fully sequenced *Treponema pallidum* Nichols strain. These proteins formed the foundation for predicting epitopes for both B-cells and T-cells. The chosen epitopes were subjected to a comprehensive assessment focusing on their stability, solubility, antigenicity, allergenicity, and toxigenicity properties. Post-selection, these epitopes were covalently conjugated with an adjuvant using specialized linkers, leading to the prediction of the vaccine’s structural model. Advanced docking simulations were subsequently performed to evaluate the binding efficacy of the vaccine with immune system components. Additional analyses were conducted to predict the immune responses elicited by the vaccine and to examine its pharmacokinetics within the host organism. In *silico* cloning methodologies were utilized to simulate the cloning process, and the resultant data were presented to facilitate the transition of the vaccine into a form suitable for protein expression.

## 2 Methods

**Figure 1:**
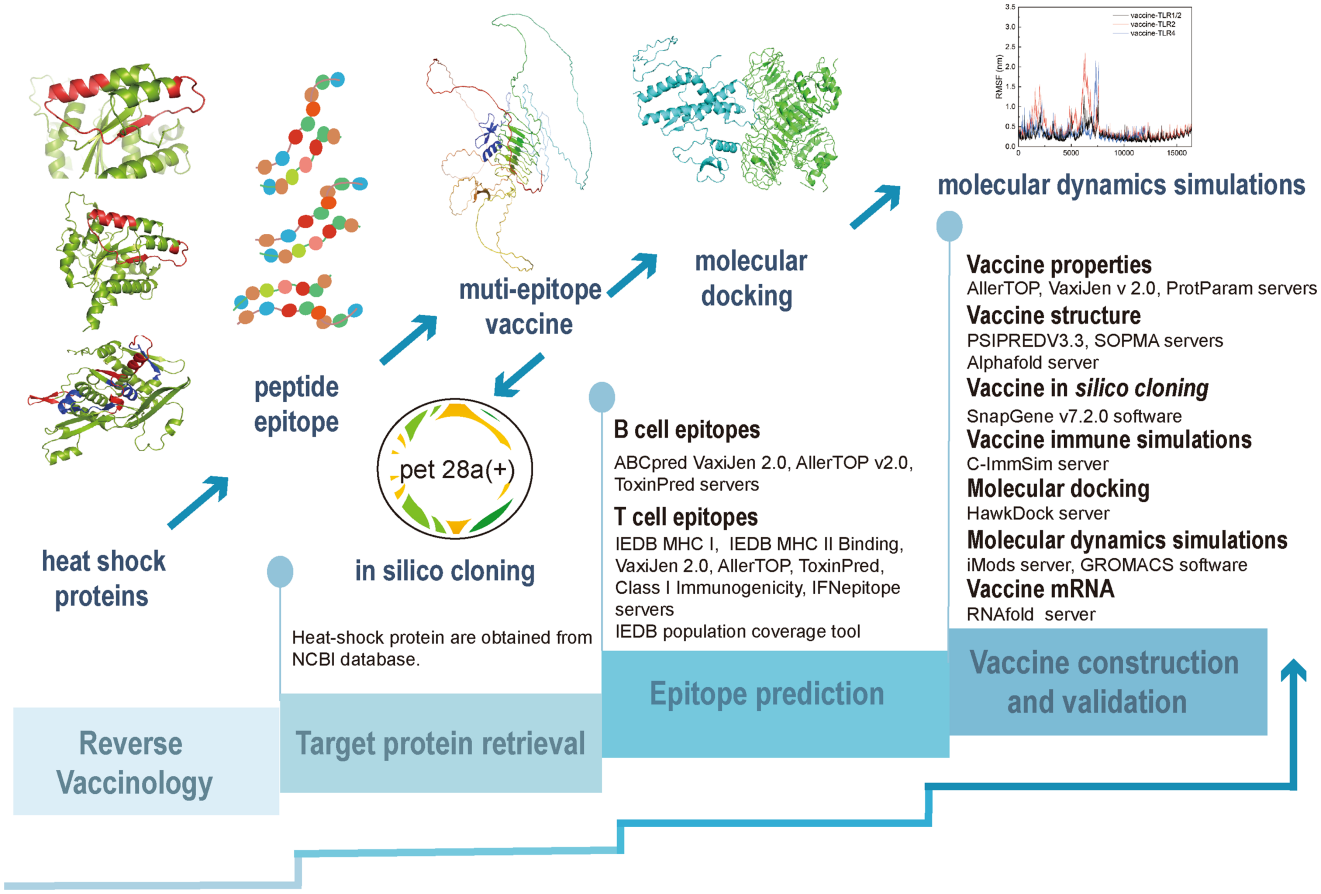
The process of development of a reverse vaccine.

In this study, we constructed a multi-epitope vaccine based on the HSPs of *T. pallidum* using the RV approach, as illustrated in Figure 1.

### 2.1 Target protein retrieval

The amino acid sequence of the heat-shock protein from *Treponema pallidum* (Nichols strain) was retrieved from the NCBI database (GenBank: AE000520.1) in FASTA format. Six heat shock proteins, namely GroEL, GrpE, DnaK, CbpA, HtpG, and DnaJ, were selected for subsequent bioinformatics analyses. These proteins were subjected to antigenicity and solubility predictions using the Vaxijen version 2.0 platform and the Protein-Sol server, respectively (Doytchinova and Flower, 2007; Hebditch et al., 2017). In an independent analysis, the FASTA sequences of these proteins were submitted to the Virulentpred online server, employing the methodologies of Cascade of Support Vector Machine (SVM) and Position-Specific Iterated BLAST (PSI-BLAST). The results generated by the Virulentpred server were based on diverse protein sequence attributes, such as amino acid composition and dipeptide composition, and demonstrated an accuracy rate of 81.8% (Garg and Gupta, 2008). We utilized the AlphaFold server to predict the three-dimensional structures of heat shock proteins. Since the evaluation results of AlphaFold2’s predictions were relatively unsatisfactory, AlphaFold3 was utilized for the tertiary structure prediction (Sahoo and Burra, 2024). The AlphaFold model has demonstrated exceptional accuracy in exploring the extensive range of biomolecular structures, underscoring its proficiency in accurately predicting the complex configurations of proteins and other biological macromolecules (Abramson et al., 2024).

### 2.2 Epitope prediction

#### 2.2.1 B cell epitopes prediction and screening

The identification of B cell epitopes, as a crucial component of multi-epitope vaccines, necessitates thorough examination. Subsequent to the submission of sequences to the ABCpred server (Saha and Raghava, 2006), assessments of antigenicity, allergenicity, and toxicity were systematically performed using the VaxiJen 2.0, AllerTOP v2.0, and ToxinPred servers, respectively (Sharma et al., 2022; Dimitrov et al., 2013). In the development of the final vaccine construct, only epitopes demonstrating robust antigenicity, non-allergenic characteristics, and non-toxicity were selected for inclusion.

#### 2.2.2 T-cell epitopes prediction

The heat shock proteins of *Treponema pallidum* were analyzed using the IEDB MHC I Binding server to predict MHC I epitopes. Concurrently, the IEDB MHC II Binding server was utilized to identify MHC II epitopes from the same heat shock proteins (Andreatta and Nielsen, 2016). These epitopes are expected to play crucial roles in inducing cytotoxic T lymphocyte (CTL) and helper T cell lymphocyte (HTL) responses, thereby initiating an adaptive immune response. The identification of CTL epitopes depends on several criteria, including the proteasomal cleavage score at the C-terminal, the efficiency of the transporter associated with antigen presentation (TAP), and the binding affinity to the major histocompatibility complex class I (MHC-I) (Larsen et al., 2007).

Moreover, the ProPred1 and ProPred servers were also employed to predict MHC I- and MHC II- binding epitopes derived from B cell epitope (Dar et al., 2019). B-cell derived T-cell epitopes could trigger both the humoral (B-Cell / Ab) and cellular (T-Cell / MHC-I/-II) immune response simultaneously. The method was elaborated in the article of Saha et al. (2021).

#### 2.2.3 MHC I and MHC II epitopes screening

Upon completing the prediction of T cell epitopes, we conducted a comprehensive evaluation of their antigenicity, allergenicity, and toxicity, adhering to the same stringent protocol utilized for B cell epitopes. Furthermore, we predicted the immunogenicity of MHC I epitopes using the Class I Immunogenicity server and assessed the IFN-inducing potential of MHC II epitopes via the IFNepitope online server (Calis et al., 2013; Dhanda et al., 2013). To predict IFN-gamma epitopes, we employed a methodology based on Support Vector Machines (SVM). Peptides that received positive scores were considered capable of inducing interferon-gamma production. For progression to the subsequent phase, only those MHC I epitopes exhibiting a positive immunogenicity score and MHC II epitopes demonstrating the ability to induce IFN were selected.

#### 2.2.4 Population coverage

We utilized the IEDB population coverage tool to perform population coverage assessments (Bui et al., 2006). Significant variability was observed in the expression levels of different Human Leukocyte Antigen (HLA) alleles across various ethnic populations globally. In designing an effective multi-epitope vaccine, it was crucial to consider the prevalence of specific HLA alleles within the global population, encompassing a wide array of ethnic groups. Population coverage estimates for class I, class II, and the combined classes were rigorously calculated.

### 2.3 Vaccine construction and validation

#### 2.3.1 Vaccine construct

Epitopes that satisfied all predefined criteria were concatenated using linker peptides to create the final vaccine construct. These linkers played a crucial role, as they not only enabled the structural assembly of the vaccine as an independent immunogen but also enhanced the production of higher antibody titers compared to isolated epitopes. Furthermore, the use of specialized peptide linkers facilitated the selection of epitopes for fusion applications. For instance, the GPGPG linker sequence was shown to effectively inhibit the formation of junctional epitopes and optimize the antigen presentation pathway, a critical process for engaging helper T lymphocyte (HTL) epitopes (Alexander et al., 1998). Simultaneously, the KK linker was specifically utilized for B cell epitopes, whereas AAY linkers were employed to interconnectcytotoxic T lymphocyte (CTL) epitopes (Rawal et al., 2021). To augment immune stimulation, an appropriate adjuvant was conjugated with EAAAK at the N-terminal of the vaccine. This integrative strategy ensured a robust and precisely targeted immune response.

As noted by Salaikumaran et al. (2022) the ranking of epitopes significantly influences the final efficacy of vaccines. We therefore randomly assigned each epitope to 20 vaccine candidates and conducted a preliminary evaluation of their relevant properties as described in Salaikumaran et al. (2022).

#### 2.3.2 Vaccine properties

The vaccine sequence was analyzed for allergenicity and antigenicity using the AllerTOP and VaxiJen v 2.0 servers, respectively. Subsequently, the ProtParam tool was utilized to predict the physicochemical properties of the sequence. Parameters such as molecular weight, theoretical isoelectric point (pI), total number of negatively and positively charged residues, instability index, aliphatic index, extinction coefficient, and the grand average of hydropathicity (GRAVY) were assessed to inform and support the experimental study. The Protein-Sol server was utilized to predict solubility in relation to protein purification processes. Additionally, the AggreProt server was employed to identify aggregation-prone regions (APRs) within protein amino acid sequences, leveraging automated deep neural network ensembles (Planas-Iglesias et al., 2024).

#### 2.3.3 Secondary and tertiary structure analysis

To determine the proportions of secondary structures in the final designed vaccine, we utilized the PSIPREDV3.3 server and the SOPMA server. The Prabi server employed the GOR4 method for two-dimensional structure prediction, while SOPMA utilized two feedforward neural networks to analyze the output generated by the Position-Specific Iterated–BLAST (PSI-BLAST) algorithm (McGuffin et al., 2000; Geourjon and Deléage, 1995). The amino acid sequence of the vaccine was submitted to the Alphafold server for three-dimensional structure prediction. Subsequently, the GalaxyRefine server was utilized to optimize and refine the tertiary structure model generated by Alphafold (Heo et al., 2013). To assess the accuracy of the three-dimensional models, we employed the ProSA-web server, a specialized analytical tool for protein structure evaluation (Wiederstein and Sippl, 2007). The server computed the Z-score, a metric wherein a value exceeding zero suggests potential defects or instabilities within the protein model. The ProSA-web Z-score provided a visual comparison between the submitted protein model and a dataset of experimental structures archived in the Protein Data Bank (PDB). Subsequently, the structural integrity of the protein was rigorously evaluated using two online platforms: PROCHECK and ERRAT. Central to this validation procedure was the Ramachandran diagram, a graphical representation used to assess the quality of the modeled three-dimensional structure by analyzing the distribution of residues across various zones: outliers, allowed, and favored (Laskowski et al., 1996). These zones correspond to different conformational states of the amino acid backbone, with the favored zone indicating the most energetically stable and commonly observed configurations. The dihedral angles psi (*ψ*) and phi (*Φ*) of the amino acids were of critical importance in this assessment. Ultimately, PyMOL software was utilized for the visualization of three-dimensional representations of the vaccine construct (Rosignoli and Paiardini, 2022).

#### 2.3.4 Structural B cell epitope prediction

The multi-epitope vaccine structure was analyzed using the ElliPro server to predict structural B cell epitopes in PDB format (Ponomarenko et al., 2008). The server assigned an elliptical score to each amino acid residue, which was quantified by the convexity index, a measure reflecting the convexity of the protein antigen’s three-dimensional conformation. Amino acids with higher convexity index values exhibited enhanced solvent accessibility and were categorized based on their radial distance (R), where an increased R value signifies a more dispersed arrangement of antigenic epitopes.

#### 2.3.5 In *silico* codon adaptation and cloning

The multi-epitope vaccine sequence was subjected to reverse transcription and subsequent codon optimization to ensure alignment with the codon usage preferences of *Escherichia coli*, thereby facilitating enhanced expression of the vaccine within the expression system. In this study, the Optimizer server was employed for reverse translation (Puigbo et al., 2007). Subsequent codon optimization for *E. coli* K12 was conducted to achieve a codon adaptation index (CAI) score ranging from 0.8 to 1, indicating a high level of alignment with the host’s preferred codon usage. Additionally, the GC content was maintained between 30 and 70%, which is considered optimal for stability and efficient translation in *E. coli* (Sharp and Li, 1987). Furthermore, to facilitate future cloning and manipulation processes, the restriction enzyme recognition sites *XhoI* and *BamHI* were selected. These sites enabled precise insertion of the gene into alternative vectors or plasmids using standard molecular biology techniques. Finally, the optimized sequence was incorporated into a cloning vector using SnapGene software version 7.2.0 (Khan et al., 2023). The software facilitated the seamless integration of the modified gene into the pET-28a(+) plasmid vector, which was sourced from the same platform. The resulting construct was then prepared for transformation into competent *E. coli* cells to enable protein expression.

#### 2.3.6 Immune simulations

To advance our comprehension of the immune response elicited by the engineered vaccine, we utilized the C-ImmSim server, a distinguished bioinformatics platform known for its proficiency in simulating and assessing the vaccine’s potential to activate a wide spectrum of immune cells. This analysis included B cells, T cells, macrophages, natural killer (NK) cells, dendritic cells, as well as the production of cytokines, which are critical mediators in immune responses. In our in *silico* simulations, we administered three distinct doses of the vaccine at four-week intervals. To ensure the accuracy of our simulations, we meticulously adjusted several parameters. The number of simulation steps was set to 1,050 on the platform. The time steps were configured as 1, 84, and 170, with each step corresponding to an actual duration of 8 h, where time step 1 represented the initial point of vaccine administration. All other parameters, including simulation volume, HLA molecules, random seed, vaccine injections, and adjuvants, were maintained at their default settings. The objective of conducting the simulation under these specified conditions was to obtain an unaltered representation of the immune response to the multi-epitope vaccine.

#### 2.3.7 Molecular docking

Molecular docking has become an advanced computational method for assessing the complex interactions between proteins and their corresponding receptors. Within the framework of inflammatory responses induced by the bacterium *Treponema pallidum*, Toll-like receptors TLR2 and TLR4 have been identified as playing crucial roles (Peng et al., 2019; Huang et al., 2020). The structures of TLR1/2 (PDB ID: 2Z81), TLR2 (PDB ID: 3A7B), and TLR4 (PDB ID: 2Z62) were retrieved from the Protein Data Bank in PDB format. The HawkDock server, which specializes in High Ambiguity Driven protein–protein DOCKing, was employed for docking studies between the vaccine and the receptor (Weng et al., 2019). This server not only leverages ambiguous interaction restraints (AIRs) but also effectively integrates unambiguous distance restraints (Honorato et al., 2021). Furthermore, the HawkDock server was utilized to perform free energy calculations of vaccine-TLR complexes using the molecular mechanics/Generalized Born surface area (MM-GBSA) methodology. This dual approach enhances the precision and reliability of the analysis, facilitating a comprehensive understanding of complex molecular systems. The binding affinity of a complex, commonly referred to as Gibbs free energy or ΔG, was determined through MM-GBSA calculations to assess the binding affinity of vaccine-TLR complexes. The PDBsum server was employed to visualize the interactions of the residues within the vaccine complexes (Laskowski et al., 2018). Additionally, the structures of the vaccine-TLR complexes were visualized using PyMol software.

#### 2.3.8 Molecular dynamics simulations

Molecular dynamics (MD) simulation is a computational method utilized to examine the temporal progression of molecular systems (Collier et al., 2020; Filipe and Loura, 2022). By probing the atomic level, MD simulations allow researchers to investigate the complex interactions and movements of atoms and molecules. In this study, MD simulations were employed to analyze the motion of atoms involved in interactions and to quantify the binding affinity of the complex vaccine-TLR assemblies. Our simulations were performed using the GROMACS (GROningen MAchine for Chemical Simulations) 2023.4 software suite, a widely utilized and user-friendly platform for molecular dynamics (MD) simulations. The initial phase of the protocol involved solvating the entire system with the simple point charge (SPC) water model, wherein the system was enclosed within a cubic boundary. This configuration provided a substantial buffer zone of 10 angstroms between the protein’s surface and the boundaries of the box. To achieve electrostatic neutrality within the system, counter-ions were subsequently added at a physiologically relevant concentration of 0.15 mol/L. Energy minimization was then conducted using the steepest descent algorithm for 5,000 iterations, with the objective of reaching a stringent force convergence criterion of less than 1,000 kcal/mol/nm. The subsequent equilibration of the Vaccine-TLR complexes was rigorously conducted using both the canonical (NVT) and isothermal-isobaric (NPT) ensembles over a period of 5 nanoseconds. This preparatory phase culminated in a 100-nanosecond molecular dynamics (MD) simulation performed under the microcanonical ensemble. Following the simulation, the trajectory files, containing coordinates recorded at 10-picosecond intervals, were thoroughly analyzed utilizing the proprietary tools available within the GROMACS suite. Finally, various statistical measures, including root-mean-square deviation (RMSD), root-mean-square fluctuation (RMSF), solvent accessible surface area (SASA), and radius of gyration (Rg), were graphically represented (Das et al., 2024).

In addition, the iMODS server was employed to conduct MD simulations *via* the normal modal analysis (NMA) algorithm for molecules collective motion (López-Blanco et al., 2014). In this online tool, we inputted Vaccine-TLR complexes in PDB format and outputted the information about deformability and B-factor.

#### 2.3.9 Vaccine mRNA prediction

The online program RNAfold was used for the prediction and evaluation of mRNA structure. After submitting the vaccine DNA sequence to this server, the mRNA secondary structure of the vaccine was then outputted in an algorithm of chemical modification (Mathews et al., 2004; Gruber et al., 2008).

## 3 Results

### 3.1 Identification of heat shock protein

**Figure 2:**
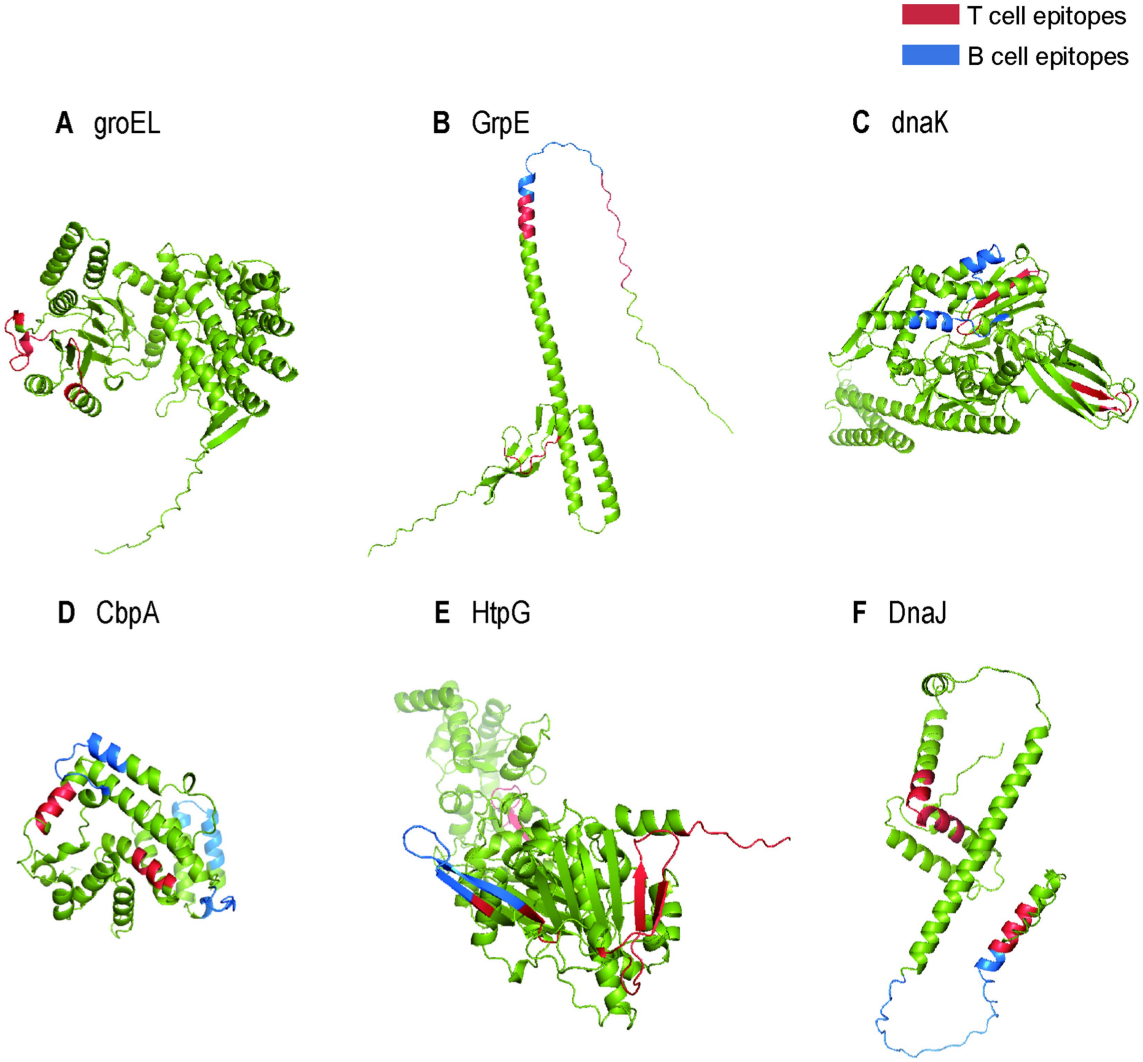
The tertiary structures of heat shock proteins. **(A)** The 3D structure of heat shock protein groEL with locus tag: TP_0030; **(B)** The 3D structure of heat shock protein GrpE with locus tag: TP_0215; **(C)** The 3D structure of heat shock protein dnaK with locus tag: TP_0216; **(D)** The 3D structure of heat shock protein CbpA with locus tag: TP_0843; **(E)** The 3D structure of heat shock protein HtpG with locus tag: TP_0984; **(F)** The 3D structure of heat shock protein DnaJ with locus tag: TP_0098. The red structures represent T cell epitopes, while the blue structures represent B cell epitopes.

The anticipated characteristics and properties of heat shock proteins are detailed in Table 1. As shown in the table, HSPs exhibit relatively common antigenicity but possesses high solubility, is nontoxic, lacks a topological helical structure and signal peptide. These characteristics provide advantages for constructing multi-epitope vaccines, making these proteins particularly favorable for vaccine expression. Furthermore, the three-dimensional structures of these proteins, highlighting the ultimately selected B cell and T cell epitopes, are illustrated in Figure 2. As illustrated in the figure, HSPs contain a rich array of T-cell and B-cell epitopes.

**Table 1:** The prediction results of heat shock proteins.

Protein	Locus_tag	Antigenicity	Solubility	Virulent	Topology	Signal peptide	Molecular weight	Instability index	Theoretical pI
groEL	TP_0030	0.4621	0.683	−1.292	0	0	57.980	24.65	5.05
GrpE	TP_0215	0.7337	0.808	−0.907	0	0	24.206	46.54	4.78
dnaK	TP_0216	0.6593	0.748	−0.944	0	0	68.041	32.67	5.05
CbpA	TP_0843	0.6824	0.306	−1.012	0	0	31.526	53.53	8.14
HtpG	TP_0984	0.3747	0.489	−1.036	0	0	72.937	38.43	5.35
DnaJ	TP_0098	0.7122	0.561	−0.031	0	0	24.122	52.77	11.04

### 3.2 Epitope prediction and screening

#### 3.2.1 Identification of B cell epitopes

The B cell epitopes of these proteins were predicted using the ABCpred epitope server. Subsequently, the antigenicity and allergenicity of the selected B-cell epitopes were evaluated. In the context of vaccine development, priority was assigned to B cell epitopes that demonstrated non-allergenic characteristics, high antigenicity, and non-toxic properties. A detailed summary of the results is provided in Table 2, where four B cell epitopes were selected.

**Table 2:** The linear B-cell epitopes of heat shock proteins.

Protein	Peptide	Position	ABCpred score	Antigenicity	Allergenicity	Toxicity
GrpE	SGEGSVPGEHSQELET	35–50	0.90	1.4378	NON-ALLERGEN	Non-Toxin
dnaK	GDTHLGGDDFDARIVQ	217–232	0.90	1.5303	NON-ALLERGEN	Non-Toxin
DnaJ	PGSIRATLLFSIWLLR	178–193	0.93	1.3506	NON-ALLERGEN	Non-Toxin
DnaJ	QERRGAPSHSGSGARP	162–177	0.90	1.5912	NON-ALLERGEN	Non-Toxin

The ABCpred score > 0.90, Antigenicity > 0.4, NON-ALLERGEN, and Non-Toxin B cell epitopes were selected.

#### 3.2.2 Identification of T cell epitopes

We conducted a comprehensive prediction of cytotoxic T lymphocyte (CTL) and helper T lymphocyte (HTL) epitopes of heat shock proteins utilizing the IEDB MHC I and MHC II binding servers. These predicted epitopes were subsequently evaluated for antigenicity, allergenicity, and toxicity to exclude those not meeting the established criteria. Following this, we calculated the inducibility of interferon-gamma (IFN-*γ*) and the immunogenicity scores for both HTL and CTL epitopes. Ultimately, a selection of seven CTL epitopes and five HTL epitopes was identified for vaccine development, as detailed in Tables 3, 4.

**Table 3:** The CTL epitopes of heat shock proteins.

Protein	Allele	Position	Peptide	Antigenicity	Immunogenicity	Allergenicity	Toxicity
groEL	HLA-B*40:01	250–258	AEDVEGEAL	1.9476	0.2855	NON-ALLERGEN	Non-Toxin
GrpE	HLA-A*68:01, HLA-B*40:01	49–58	ETGASEETLR	1.9681	0.1073	NON-ALLERGEN	Non-Toxin
CbpA	HLA-A*24:02, HLA-A*23:01	89–97	KFDLLYARF	1.7882	0.04502	NON-ALLERGEN	Non-Toxin
CbpA	HLA-A*31:01	256–264	RTGDRRRAR	2.6559	0.19898	NON-ALLERGEN	Non-Toxin
HtpG	HLA-A*02:06, HLA-A*02:01 HLA-A*02:03, HLA-B*15:01	2–10	AQYEFQTEV	1.1520	0.19577	NON-ALLERGEN	Non-Toxin
DnaJ	HLA-A*31:01, HLA-A*02:01	185–193	LLFSIWLLR	2.4222	0.1938	NON-ALLERGEN	Non-Toxin
DnaJ	HLA-A*01:01, HLA-A*31:01	60–69	LSDRASRARY	1.1808	0.04826	NON-ALLERGEN	Non-Toxin

The Antigenicity > 0.4, Immunogenicity > 0, NON-ALLERGEN, Non-Toxin CTL epitopes were selected.

**Table 4:** The HTL epitopes of heat shock proteins.

Protein	Allele	Position	Peptide	Adjusted rank	IFN-g scores	Antigenicity	Allergenicity	Toxicity
groEL	HLA-DRB4*01:01	303–317	DLGLKLESADIALLG	0.43	0.3716	1.0543	NON-ALLERGEN	Non-Toxin
GrpE	HLA-DRB1*07:01, HLA-DRB1*09:01	22–36	ADSLRASDPVPVESG	0.32	0.1320	0.8922	NON-ALLERGEN	Non-Toxin
HtpG	HLA-DRB1*11:01 HLA-DRB1*08:02 HLA-DRB1*13:02	490–504	EWELRAINRLGSEEE	0.02	0.1839	1.0357	NON-ALLERGEN	Non-Toxin
HtpG	HLA-DRB3*01:01	220–234	QKEYDKDGAVTDTQK	0.4	0.009	1.2387	NON-ALLERGEN	Non-Toxin
DnaJ	HLA-DQA1*04:01/DQB1*04:02	54–68	NAAYAVLSDRASRAR	0.31	0.8966	0.8197	NON-ALLERGEN	Non-Toxin

The Adjusted rank < 0.5, IFN-g scores > 0, Antigenicity > 0.4, NON-ALLERGEN, Non-Toxin HTL epitopes were selected.

The B-cell derived T-cell epitopes was displayed in Supplementary Tables 1, 2 as a reference.

#### 3.2.3 Population coverage of T cell epitopes

**Figure 3:**
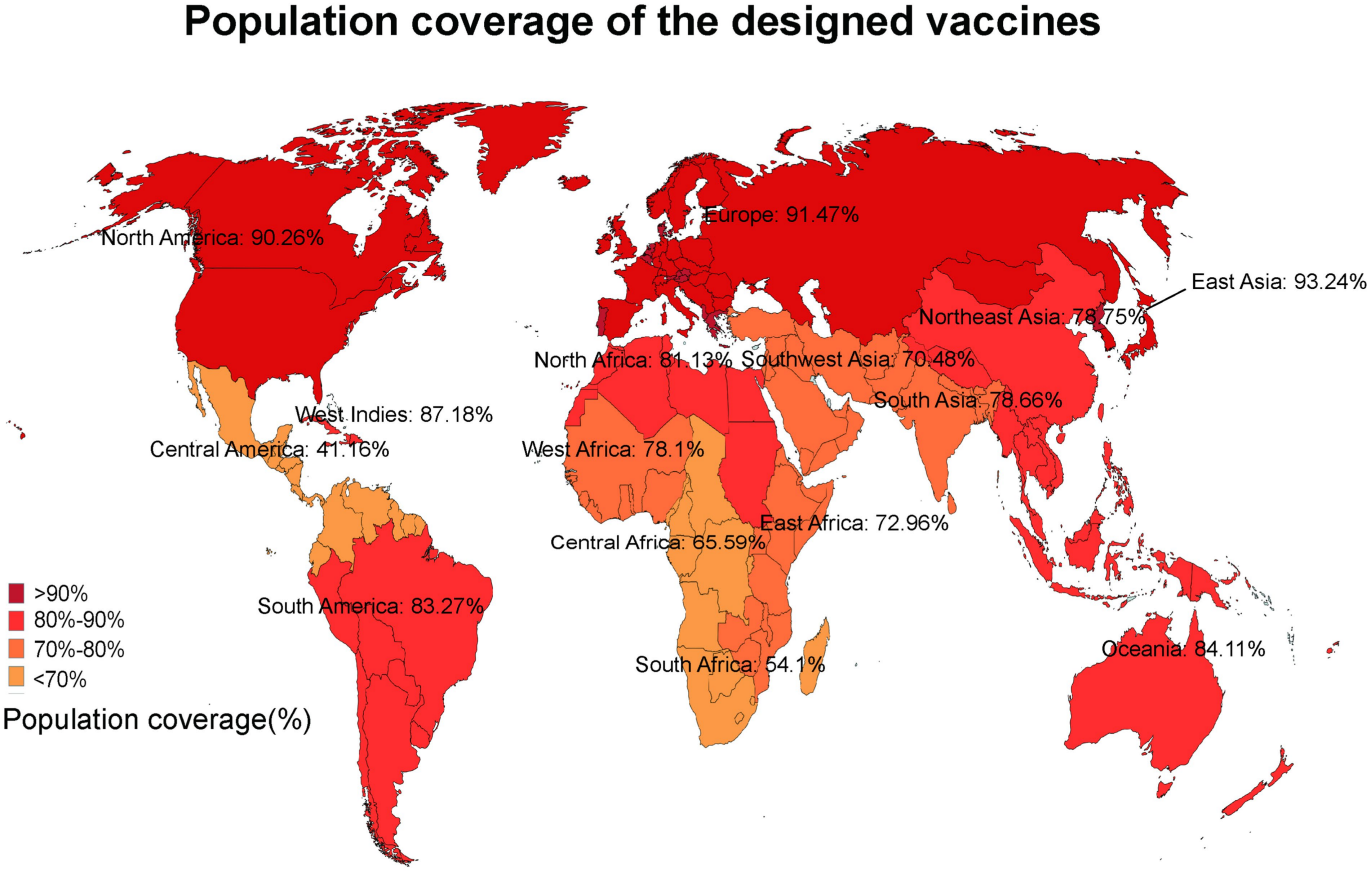
The population coverage of MHC I and MHC II epitopes worldwide.

The anticipated T cell coverage was derived from the inclusion of both MHC I and MHC II epitopes within the vaccine protein. According to the IEDB population coverage tool, the combined population coverage of MHC I and MHC II epitopes varied between 34.9 and 94.41% across 17 distinct geographical regions, as illustrated in Figure 3. The following regions were chosen: World (94.41%), East Asia (93.24%), Northeast Asia (78.75%), South Asia (78.66%), Southeast Asia (83.76%), Southwest Asia (70.48%), Europe (91.47%), East Africa (72.96%), West Africa (78.1%), Central Africa (65.59%), North Africa (81.13%), South Africa (54.1%), West Indies (87.18%), North America (90.26%), Central America (41.16%), South America (83.27%), Oceania (84.11%).

### 3.3 Construction of epitope-based vaccine

#### 3.3.1 Multi-epitope vaccine construct

The ultimate vaccine design comprised epitopes, specifically four B cell epitopes linked via the KK linker, seven cytotoxic T lymphocyte (CTL) epitopes linked using the AAY linker, and five helper T lymphocyte (HTL) epitopes linked through the GPGPG linker. The integration of these linkers was essential to ensure optimal amino acid residue versatility, thereby facilitating appropriate protein folding or sustaining an extended conformation. To augment the immune response and enhance epitope efficacy, the N-terminal region of the vaccine structure was conjugated to *β*-defensin using an EAAAK linker. The antigenicity of the constructed vaccine was determined to be 1.0843 using the Vaxijen server, and it was identified as non-allergenic according to the AllerTOP server. Enhanced solubility is often indicative of a more efficient purification process during the advanced stages of production. The solubility value was measured at 0.605 using the Protein-Sol server, with values exceeding 0.45 suggesting greater solubility compared to typical soluble *E. coli* proteins. Additionally, the AggreProt server indicated low aggregation propensity for the vaccine. Furthermore, analysis with the ProtParam server revealed that the final vaccine comprised 502 amino acids. The final vaccine exhibited a molecular weight of 54.23 kDa, classifying it as a small-scale construct that facilitated ease of handling and purification during testing. The theoretical isoelectric point (pI) was calculated to be 9.48, with the protein comprising 62 negatively charged and 86 positively charged amino acid residues. The instability index of the constructed vaccine was determined to be 33.20, indicating a stable protein. Furthermore, the aliphatic index, which is indicative of thermal stability, was calculated to be 66.45, suggesting that the vaccine is thermally stable. The grand average of hydropathicity (GRAVY) was found to be −0.687.

In Supplementary 4, we assessed 20 vaccines built by random epitopes ranking. It demonstrating that the properties of our constructed vaccines remained largely unaffected by changes in epitope ordering. To further demonstrate this, we developed new vaccines based on the B-cell derived T-cell epitopes and used the same method to predict the properties of 20 vaccine candidates, each composed of distinct epitope ranking. As shown in Supplementary 5, the properties of the vaccine we constructed remained largely unchanged following the alteration in epitope ranking. Therefore, we continued to use the initial vaccine for subsequent analysis.

#### 3.3.2 Secondary structure prediction

**Figure 4:**
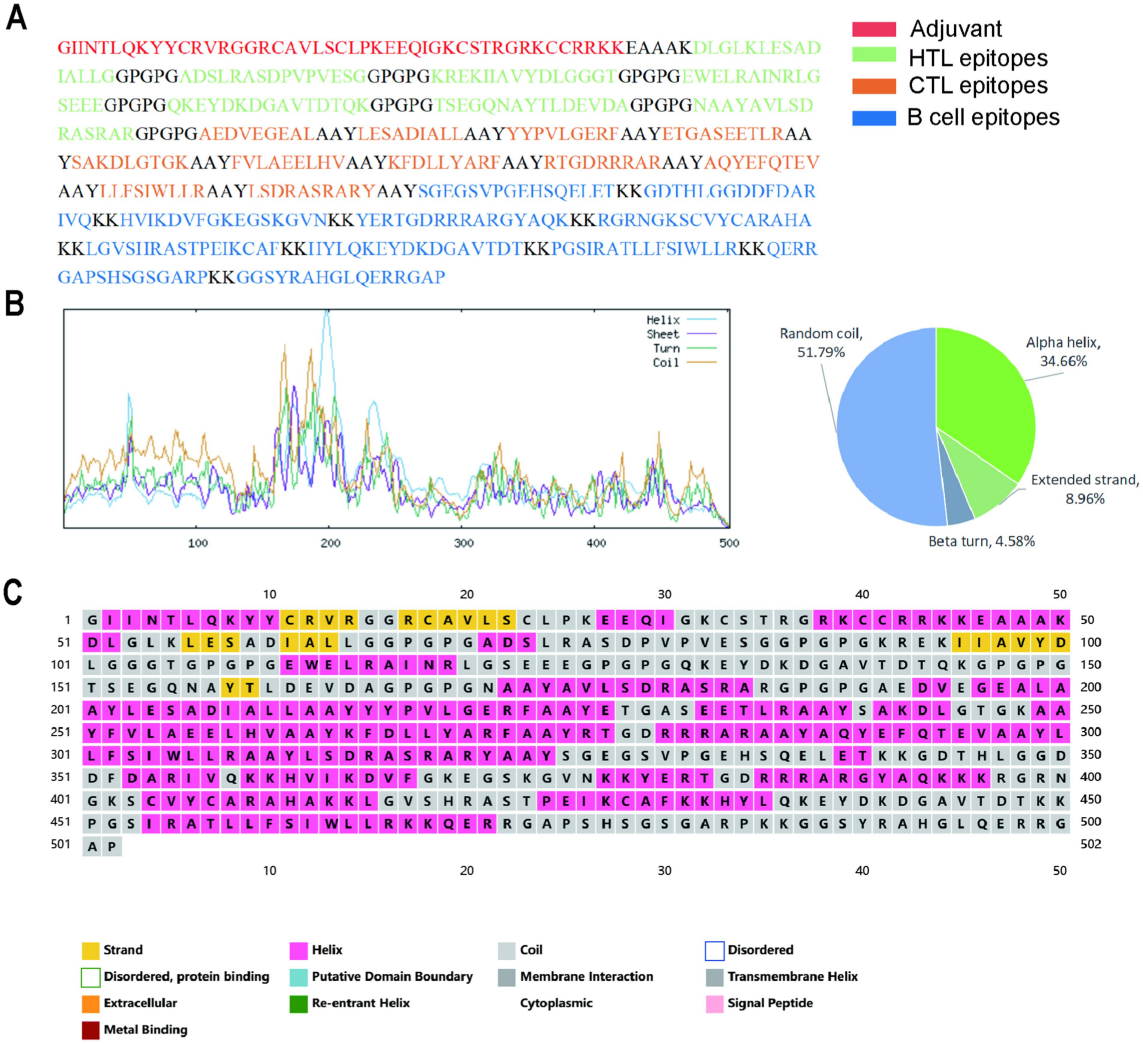
The secondary structure model of the designed vaccine. **(A)** The amino acid composition of the vaccines; **(B)** The secondary structure prediction results of SOPMA server; **(C)** The secondary structure prediction results of PSIPREDV3.3 server.

The secondary structural characteristics of the constructed vaccine, as analyzed by the SOPMA and PSIPREDV3.3 servers, are presented in Figure 4. The sequence comprises 34.66% alpha helices, 8.96% extended strands, 4.58% beta turns, and 51.79% random coils.

#### 3.3.3 Tertiary structure prediction

**Figure 5:**
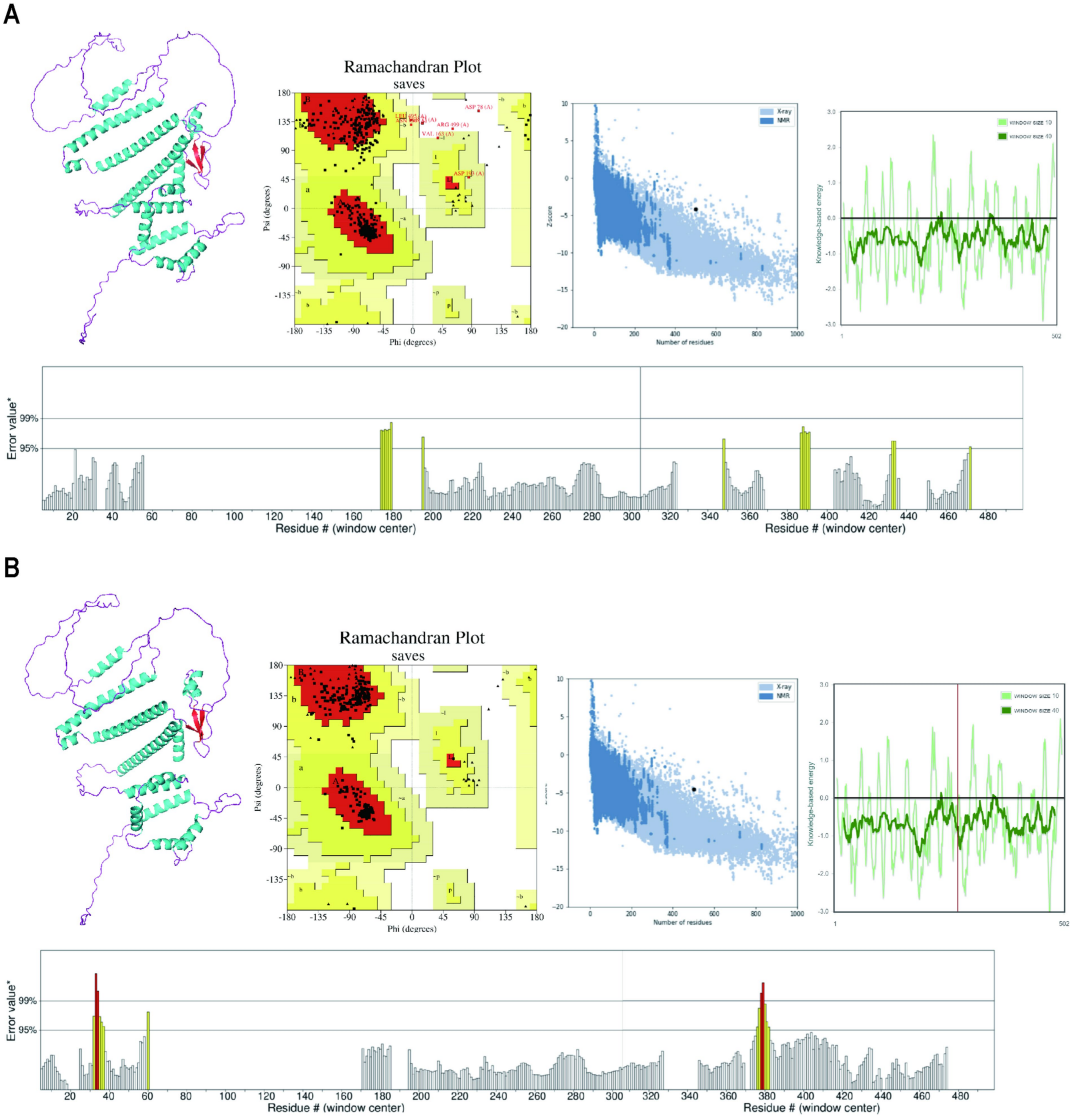
The prediction and refinement of the tertiary structure model. **(A)** The 3D structure, PROCHECK Ramachandran plot, ProSA graphical plot and ERRAT overall quality of the Alphafold model. **(B)** The 3D structure, PROCHECK Ramachandran plot, ProSA graphical plot and ERRAT overall quality of the GalaxyreWEB model.

Following the prediction by the Alphafold server, the tertiary structure model was submitted to the GalaxyWEB server for structural refinement. The tertiary structures were visualized using PyMol software. Subsequently, both the Alphafold model (Figure 5A) and the GalaxyWEB model (Figure 5B) underwent validation using the PROCHECK, ProSA-web, and ERRAT servers. The analysis conducted using the PROCHECK server revealed that the optimized three-dimensional structure obtained from the GalaxyreWEB server comprised 88.2% core, 10.1% allowed, 1.0% generously allowed, and 0.7% disallowed regions. This represents a notable structural enhancement compared to the original structure generated by the AlphaFold server, which consisted of 98.1% core, 1.9% allowed, and no generously allowed or disallowed regions. Furthermore, the Z-score from the ProSA-web server improved from −4.16 to −4.55, both of which exceed the average Z-score for comparable natural proteins. Additionally, the ERRAT server score increased from 93.84 to 95.63, indicating further optimization.

#### 3.3.4 Structural B cell epitope prediction

**Figure 6:**
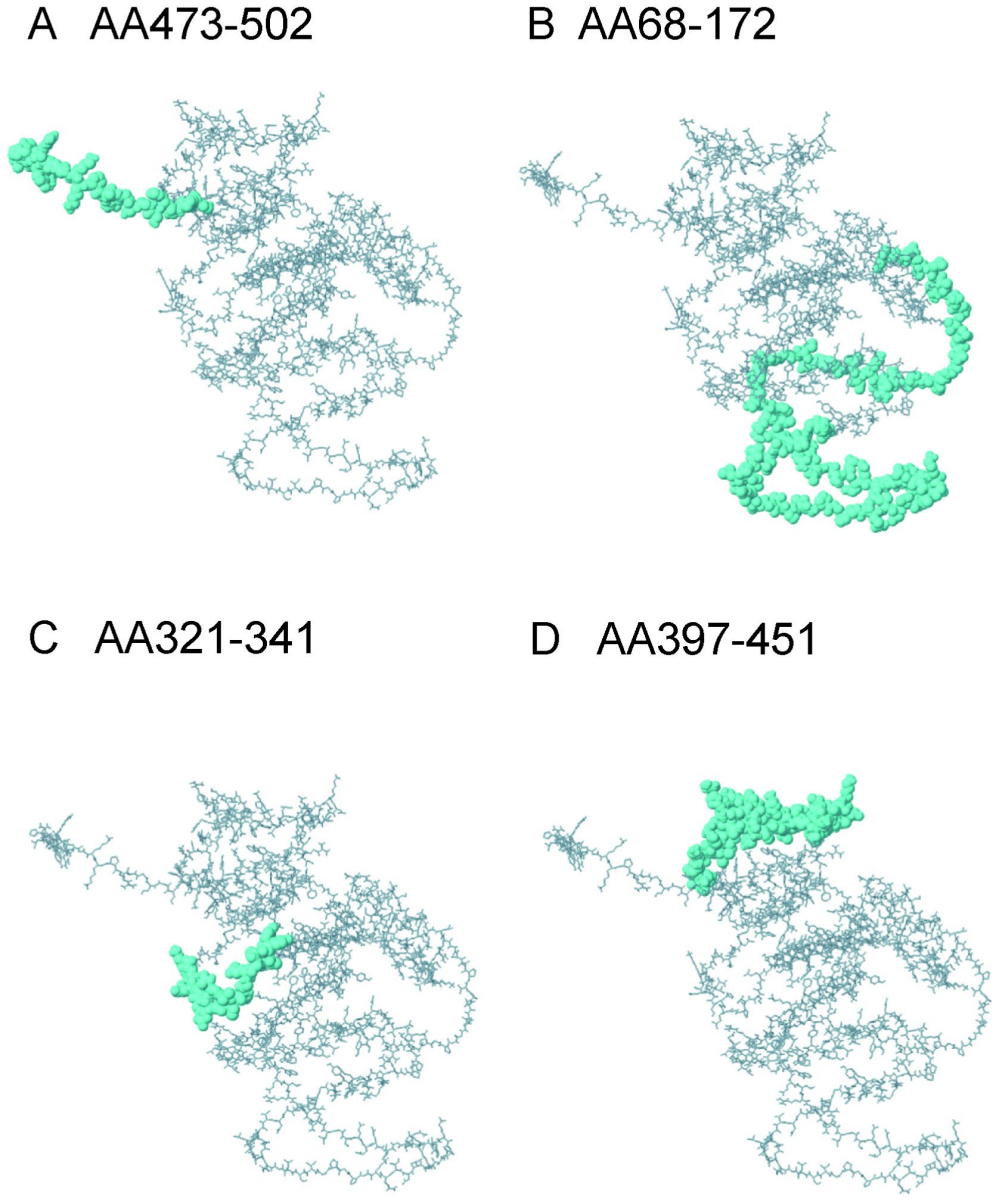
The linear B cell epitopes in the ElliPro server. **(A)** AA473-502: GAPSHSGSGARPKKGG SYRAHGLQERRGAP, ElliPro score:0.915; **(B)** AA68-172: GPGADSLRASDPVPVESGGPGPGKREKIIA VYDLGGGTGPGPGEWELRAINRLGSEEEGPGPGQKEYDKDGAVTDTQKGPGPGTSEGQNAYTLDEVDAGPGPGNA ElliPro score:0.825; **(C)** AA321-341: YAAYSGEGSVPGEHSQELETK ElliPro score:0.644; **(D)** AA397-451: RGRNGKSCVYCARAHAKKLGVSHRASTPEIKCAFKKHYLQKEYDKDGAVTDTKKP ElliPro score:0.642.

The ElliPro server identified nine structural linear B cell epitopes within the constructed vaccine protein. Figure 6 illustrates the four epitopes with the highest scores. The scores for all linear conformational B cell epitopes ranged from 0.508 to 0.915.

#### 3.3.5 Vaccine in *silico* cloning

**Figure 7:**
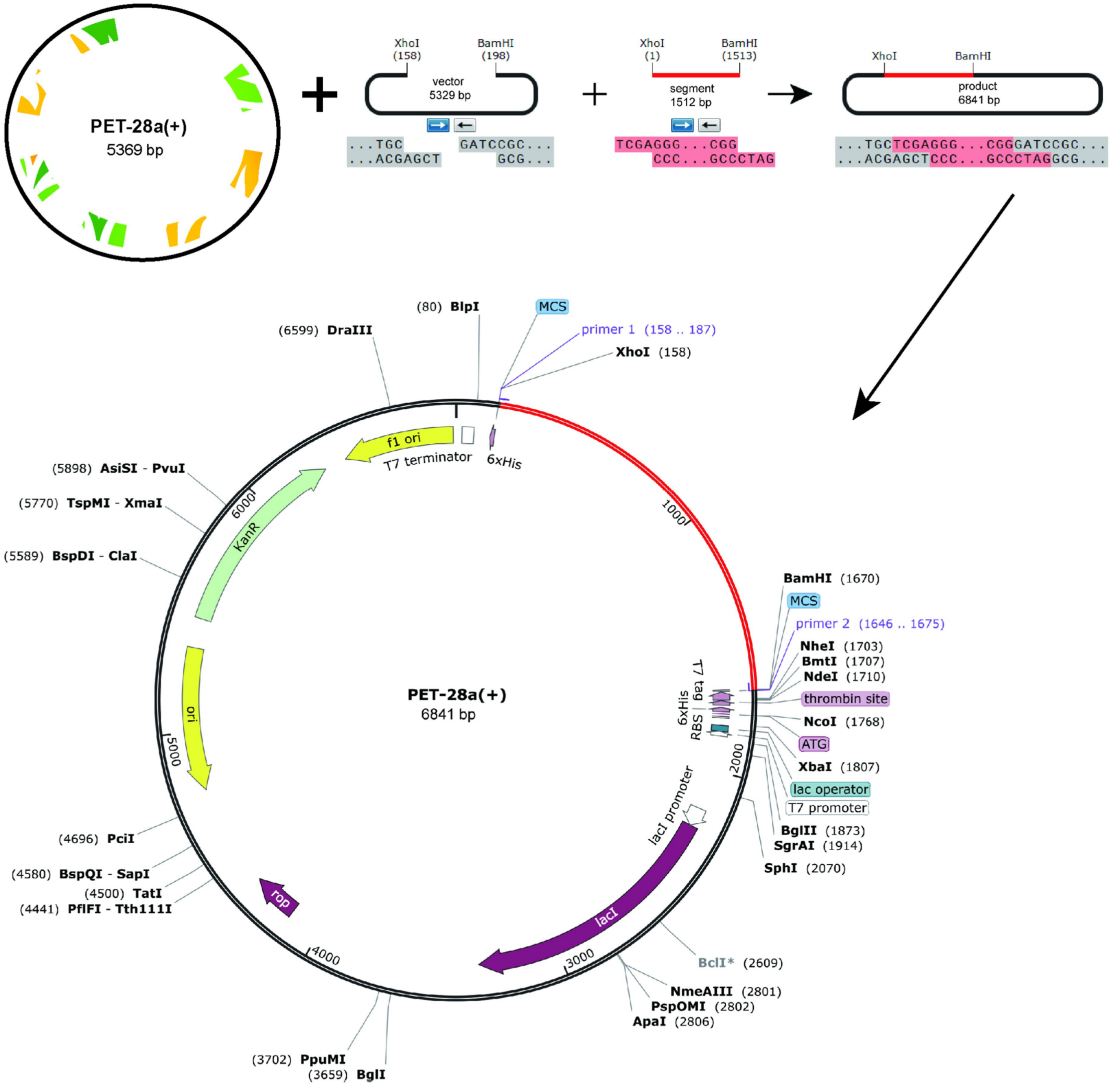
In *silico* restriction, the final vaccine construct into the pET28a (+) expression vector by cloning, where the red area represents the vaccine insert and the black circle represents the vector.

The protein sequence of the vaccine was submitted to the Optimizer server for codon adaptation specific to the *E. coli* K12 strain. The resulting codon sequence of the vaccine comprised 1,506 nucleotides. The GC content of the optimized sequence was 57.5%, and the Codon Adaptation Index (CAI) was calculated to be 1. The termini of the vaccine gene were modified to include two restriction sites, *XhoI* and *BamHI*. Subsequently, SnapGene software was employed to insert the vaccine gene into the restriction site of the pET28a(+) plasmid, as illustrated in Figure 7. The total length of the resulting clone was 6,841 base pairs.

#### 3.3.6 Immune simulations

**Figure 8:**
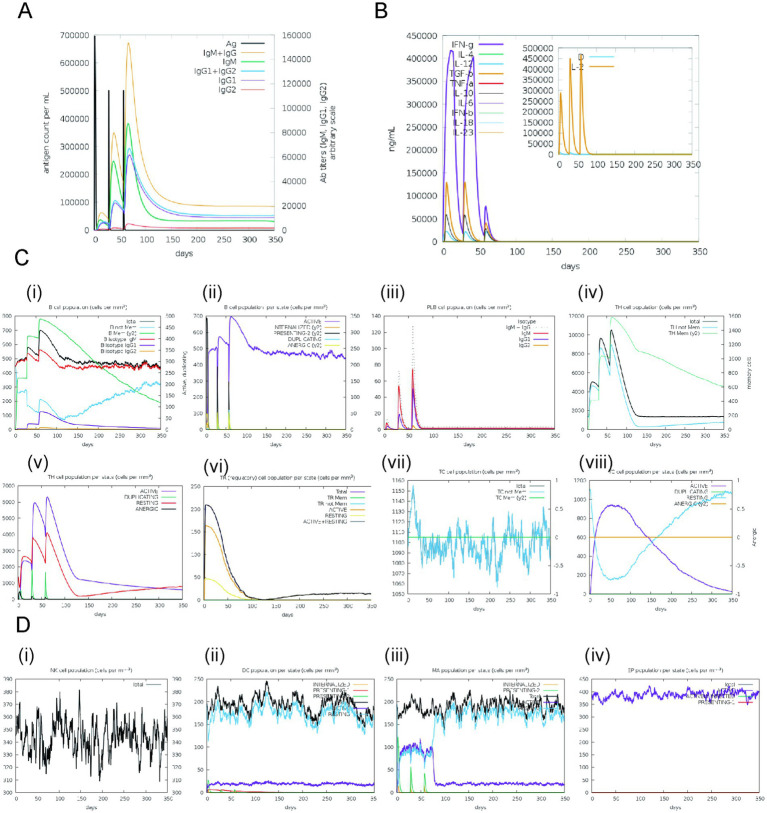
Simulated immune responses generated in response to the designed vaccine. **(A)** Immunoglobulin responses with respect to the vaccine exposure, **(B)** Cytokine responses, **(C)** (i)–(iii) Bcell responses, (iv)–(viii) T-cell responses **(D)** (i) Natural Killer cells responses, (ii) Dendritic cells responses, (iii) Macrophages responses, (iv) Epithelial cells responses.

The simulated immune responses to pathogens demonstrated remarkable fidelity to actual biological processes within the C-ImmSim server, as evidenced in Figure 8. Specifically, Figure 8A reveals that the concentrations of specific antibodies, namely IgM, IgG1, and IgG2, increased significantly during secondary and tertiary immune responses, which coincided with a reduction in antigen levels. A similar pattern of memory cell proliferation was observed among T helper and cytotoxic T cell populations, which were instrumental in augmenting immune responses, as illustrated in Figure 8C. Furthermore, a notable enhancement in macrophage activity and interactions was observed, alongside an expansion of dendritic cells, as illustrated in Figure 8D. Elevated levels of cytokines, such as interferon-gamma (IFN-*γ*) and interleukin-2 (IL-2), as shown in Figure 8B, were associated with increased risks during immune responses, as indicated by the Simpson Index D. These findings collectively underscore the intricate dynamics and adaptive capabilities of the human immune system in its defense against pathogens.

#### 3.3.7 Molecular docking

**Figure 9:**
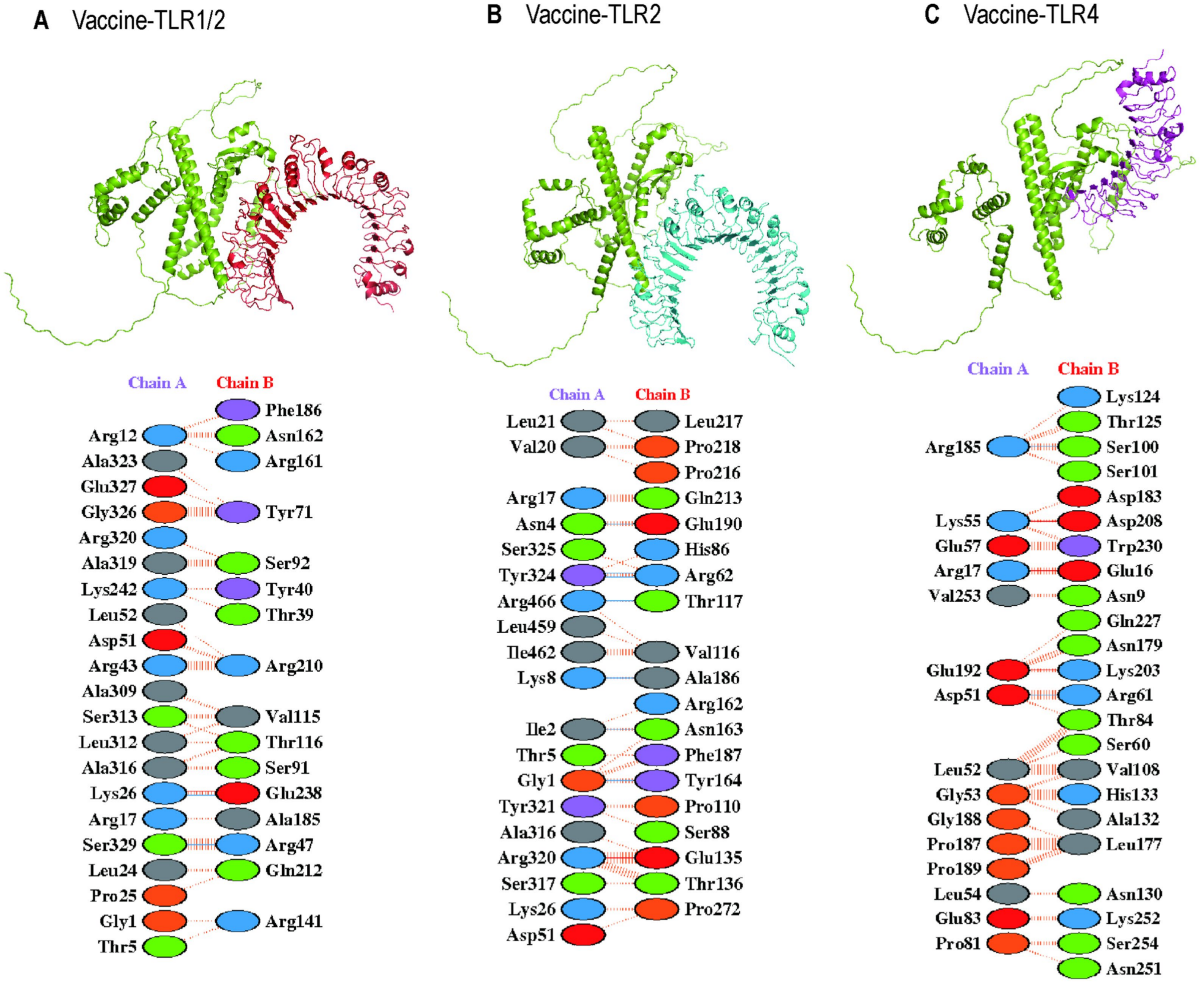
The molecular docking structure in the HawkDock server and interaction in the PDBsum server. **(A)** The 3D structure and interaction of vaccine-TLR 1-2 complex; **(B)** The 3D structure and interaction of vaccine-TLR 2 complex; **(C)** The 3D structure and interaction of vaccine-TLR 4 complex.

TLR2 and TLR4 were chosen as the immune receptors due to their critical role in the immune response against *T. pallidum*. Molecular docking analyses were performed between the multi-epitope vaccine and the immune receptors TLR1/2 (PDB ID: 2Z81), TLR2 (PDB ID: 3A7B), and TLR4 (PDB ID: 2Z62). The HawkDock server facilitated the molecular docking and binding free energy calculations. The HawkDock scores for the vaccine-TLR complexes were −6542.19, −7214.02, and −6664.40, respectively. Figure 9 presents the molecular docking structures and interchain interactions as visualized using the PDBsum server. The total binding energy (ΔG_TOTAL_) of vaccine-TLR1/2, vaccine-TLR2, and vaccine-TLR4 were −22.52 kcal/mol, −30.69 kcal/mol, and −32.07 kcal/mol, respectively. The binding free energy consists of Vander Waals energy (ΔE_vdW_), electrostatic energy (ΔE_elec_), polar solvation energy (ΔG_GB_), and non-polar solvation energy (ΔG_SA_). The specific values are shown in Table 5.

**Table 5:** The MM-GBSA free energies and individual free energy components of constructed vaccine-TLR complexes.

Energy component	MMGBSA (kcal/mol)		
	Vaccine–TLR1-2	Vaccine–TLR2	Vaccine–TLR4
ΔE_VDW_	−95.88	−90.14	−90.47
ΔE_elec_	−1309.17	−1606.7	−1397.19
ΔG_GB_	1395.05	1678.58	1467.82
ΔG_SA_	−12.52	−12.44	−12.23
ΔG_TOTAL_	−22.52	−30.69	−32.07

#### 3.3.8 Molecular dynamics simulations

**Figure 10:**
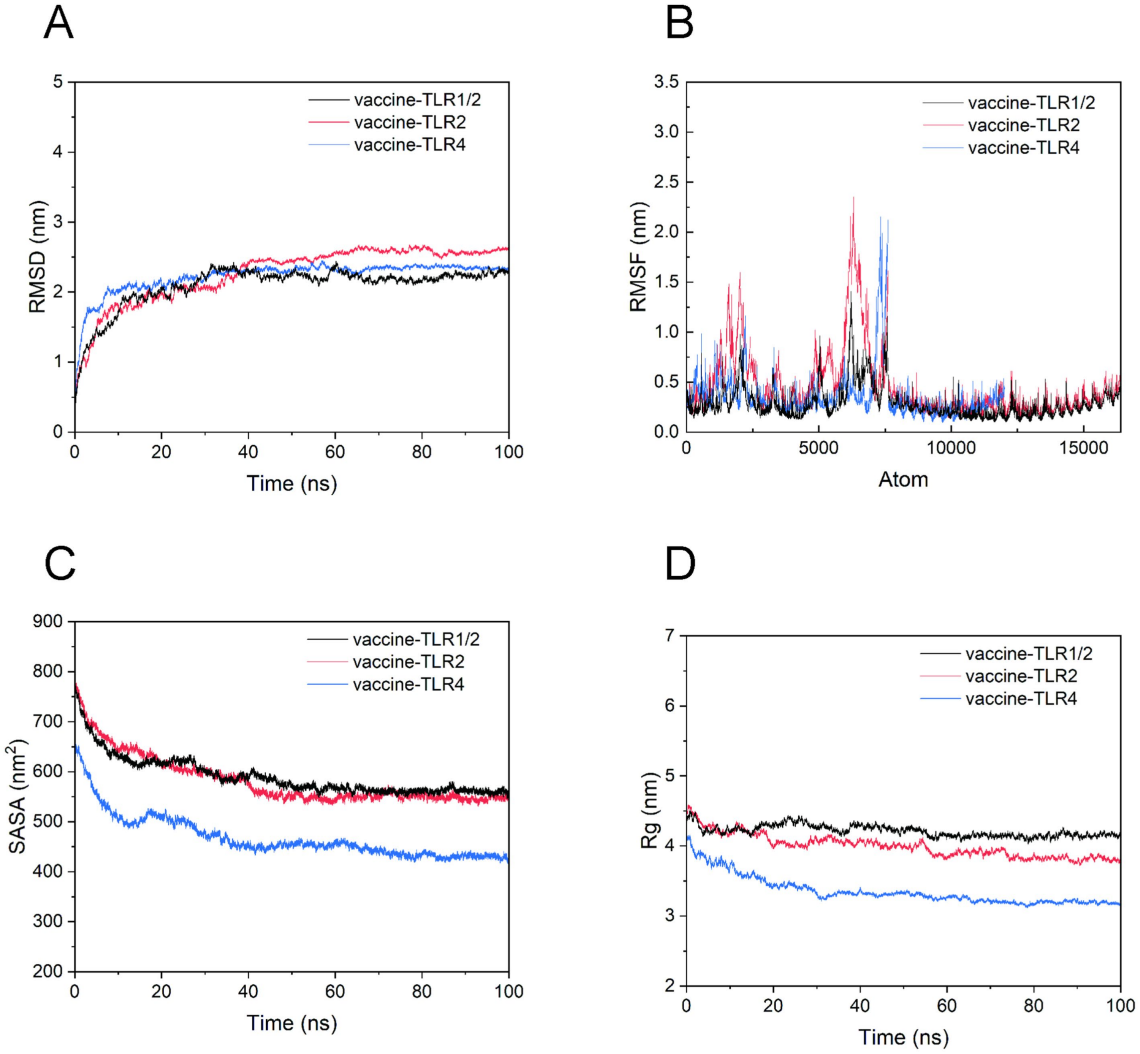
MD simulation elucidating conformational stability of the interactions between vaccine and TLRs. **(A)** RMSD plot, comparing the stability of vaccine-TLR complexes; **(B)** RMSF plot, confirming the flexibility of vaccine-TLR complexes; **(C)** SASA plots, illustrating the compaction of the vaccine-TLR complexes; **(D)** Rg plot, indicating compactness of vaccine-TLR complexes.

Molecular dynamics (MD) simulation offers a detailed, temporal insight into system dynamics over a broad range, enabling a comparative assessment of the conformational integrity of the three vaccine-TLR complexes. A comprehensive analysis of the MD simulations is presented in Figure 10. With respect to stability, the RMSD plot indicates that all three vaccine-TLR complexes maintain conformational stability. However, the average RMSD obtained during the simulation suggests that the vaccine-TLR2 complex exhibits slightly greater stability compared to the other two complexes. Furthermore, the vaccine-TLR1/2, vaccine-TLR2, and vaccine-TLR4 complexes achieved stability after a 10 ns time step and maintained this stability up to the 100 ns mark. The Root Mean Square Fluctuation (RMSF) analysis determines the flexibility or oscillatory behavior of individual residues within a biomolecular system. Enhanced oscillations observed in the RMSF plot indicate an increased presence of loop regions within the system. Conversely, the presence of helices and sheets contributes to structural rigidity. As illustrated in Figure 10B, the vaccine-TLR1/2 complex exhibits greater stability compared to other complexes, as evidenced by an average RMSF value remaining below 1.5 nm. Moreover, the presence of hinges with RMSF values not exceeding 0.8 nm contributes to the overall flexibility of the vaccine-TLR1/2 complex. In contrast, the other two complexes exhibit a wider range of RMSF values, varying approximately between 2.4 and 0.2 nm for the vaccine-TLR2 complex and 2.2–0.1 nm for the vaccine-TLR4 complex, indicating a comparatively higher level of instability.

SASA measurements offer valuable insights into the accessibility of a biomolecule’s surface to solvent molecules. Variations in SASA are indicative of conformational changes and structural compaction occurring during molecular dynamics (MD) simulations. Elevated SASA values suggest dynamic behavior within flexible loops or disordered regions of the biomolecule. As illustrated in Figure 10C, the three complexes—vaccine-TLR1/2, vaccine-TLR2, and vaccine-TLR4—exhibit significant SASA values, thereby affirming their folded conformations.

The evaluation of the compactness of the molecular docking complex is conducted through the analysis of the radius of gyration (Rg), which offers a comprehensive insight into the biomolecules’ shape, folding, and structural stability by calculating the root mean square distance of each atom from the center of mass. A decrease in Rg signifies a more compact structure. As illustrated in Figure 10D, among the three complexes analyzed, the vaccine-TLR4 complex demonstrates the most compact configuration, with an average Rg of less than 4 nm. Nonetheless, regarding compactness, the other two complexes also displayed substantial Rg values, affirming their structural integrity as well.

**Figure 11:**
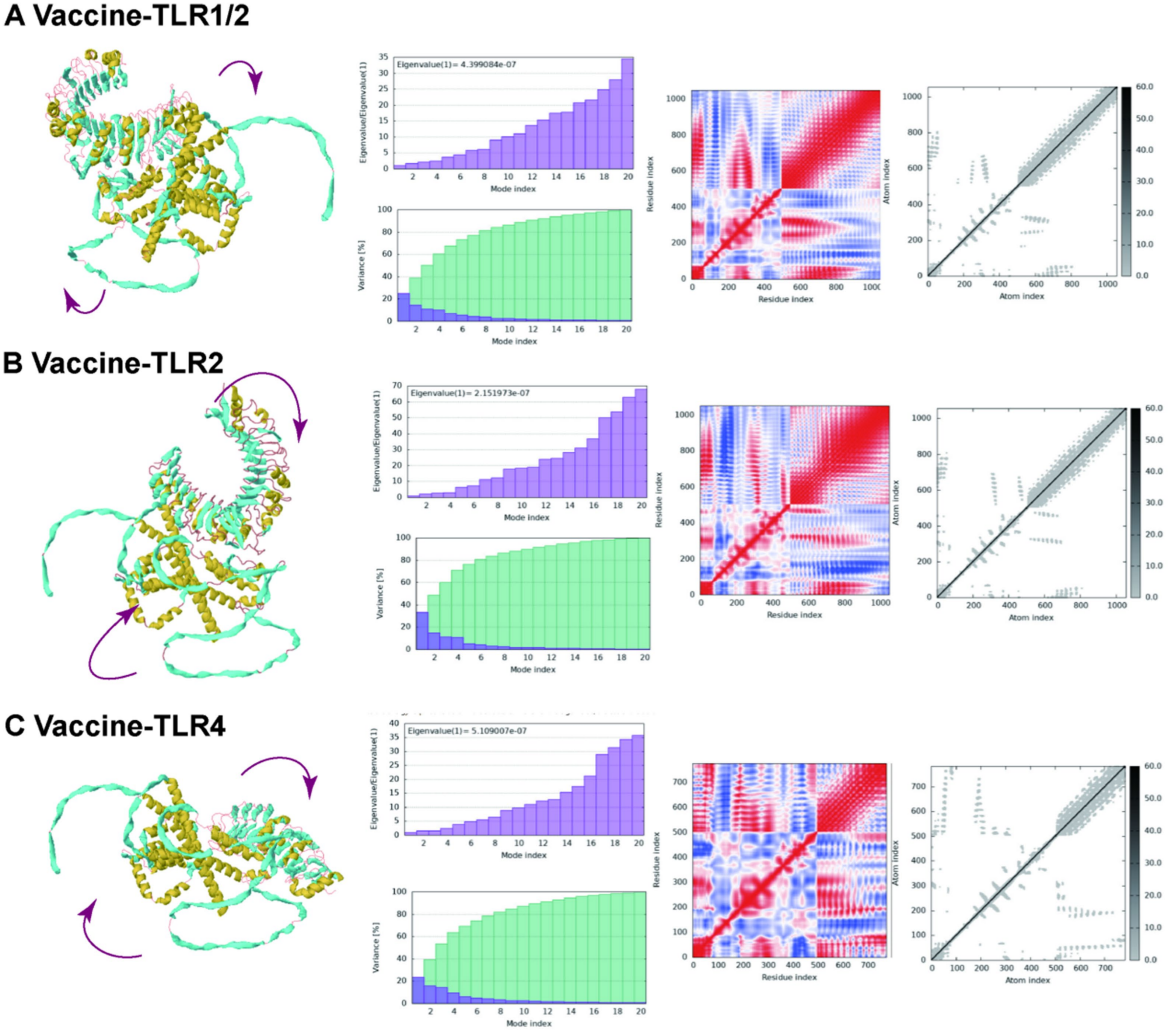
Molecular dynamics simulation of the vaccine-TLR complexes in the iMODS server. **(A)** Molecular dynamics simulation of the vaccine-TLR 1-2; **(B)** Molecular dynamics simulation of the vaccine-TLR 2; **(C)** Molecular dynamics simulation of the vaccine-TLR 4.

The results from the iMODS website are illustrated in Figure 11. This figure includes various plots: mobility, eigenvalues, variance, covariance, and elastic networks. The eigenvalues plots, depicted in purple, are directly associated with the energy required to deform the structure; lower eigenvalues indicate that carbon atoms can deform more readily. Specifically, the eigenvalues for vaccine-TLR1/2, vaccine-TLR2, and vaccine-TLR4 were 4.399084e-05, 2.151973e-05, and 5.109007e-05, respectively. In the variance plots, the green segments represent cumulative variance, while the purple segments illustrate individual variance. The covariance plot uses red to denote correlated motion, white for non-correlated motion, and blue for anti-correlated motion. Within the elastic networks, lighter dots signify flexible regions, whereas darker areas indicate stiffer sections.

#### 3.3.9 Vaccine mRNA prediction

**Figure 12:**
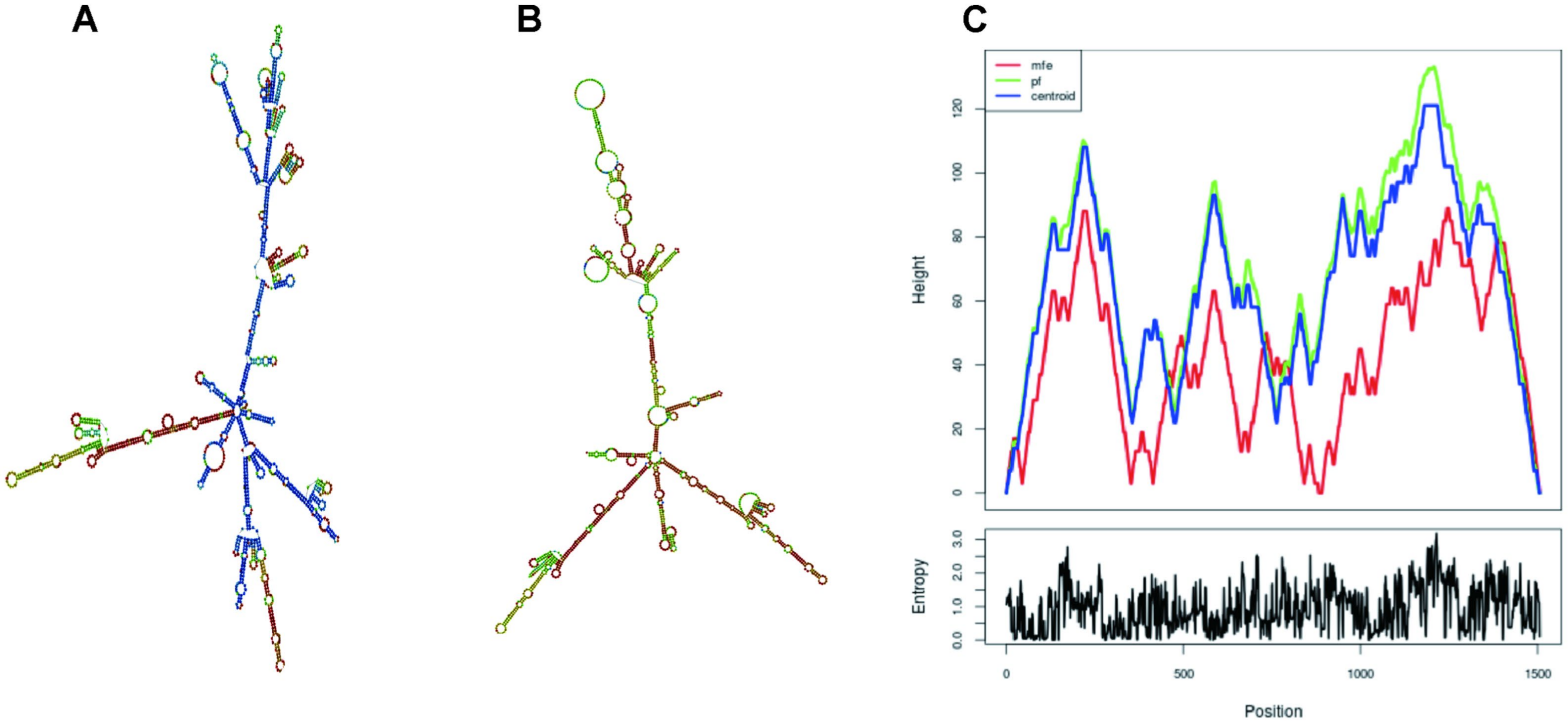
The secondary structure of vaccine mRNA. **(A)** MFE structure; **(B)** Centroid secondary structure (Colors representing base-pair probability); **(C)** The mountain plot illustrates the correlation between the MFE structure, centroid structure, and thermodynamic ensemble of RNA structure.

The optimized DNA sequence by the Optimizer website was then uploading to the online tool RNAfold for mRNA structure and structure minimum free energy (MFE) prediction (Figure 12). The MFE of optimal and centroid secondary structure are −599.00 kcal/mol and −522.47 kcal/mol, respectively. The free energy of the thermodynamic ensemble is −623.92 kcal/mol. The more negative the free energy coefficient, the more stable and long-lasting the vaccine mRNA formed within the body.

## 4 Discussion

### 4.1 Mian interpretation

*Treponema pallidum* is distinguished as the most virulent subspecies of the syphilis spirochete, primarily due to its capacity to consistently cross both the blood–brain barrier and the maternal-fetal placental barrier (Radolf et al., 2016). The transmission of venereal syphilis occurs when treponemes are introduced to mucosal surfaces or the skin through sexual contact. These spirochetes either directly penetrate mucous membranes or enter through disruptions in the skin during sexual activities (Peeling et al., 2017). Although penicillin remains an effective treatment, the incidence of syphilis has increased in recent years, with recurrent infections being relatively common. Under these circumstances, the development of an effective vaccine represents the most promising strategy for controlling and preventing the spread of this elusive pathogen. Recently, researchers have made significant advancements in the development of novel vaccines targeting *T. pallidum* (Rawal et al., 2021; Lukehart et al., 2022; Xu et al., 2021). However, there are currently no commercially available vaccines, which are mostly in the experimental stage. To deep investigation the vaccine efficacy, the experimental animal model of C57BL/6 mice was established as a convenient way in recent research (Weng et al., 2019).

Heat shock proteins (Hsps) are evolutionarily conserved molecules that play a critical role in facilitating the proper folding of other proteins. The majority of Hsps are inducible under stress conditions, reflecting their essential cytoprotective functions in cells experiencing environmental stress (Zininga et al., 2018). Notably, specific Hsp family members, such as Hsp60, Hsp70 (Milani et al., 2002), and gp96, exhibit dual functionality, serving as both pro-inflammatory and anti-inflammatory mediators (Zachova et al., 2016). Hsps have the potential to act as indicators, drivers, or modulators of various pathological processes, making them promising candidates for biomarker identification (Pockley and Henderson, 2018). Their application also extends to the development of microbial vaccines (Padula et al., 2023), as demonstrated by their use in addressing pathogens such as *Cryptosporidium hominis* (Pandya and Kumar, 2023a) and *Schistosoma mansoni* (Pandya and Kumar, 2023b). In our research, we utilized the heat shock protein of *T. pallidum* to develop a vaccine, employing an epitope-based strategy to enhance its efficacy.

To facilitate the development of a multi-epitope vaccine, six heat shock proteins were identified as potential candidates: groEL (locus tag: TP_0030), GrpE (locus tag: TP_0215), dnaK (heat shock protein 70, locus tag: TP_0216), CbpA (locus tag: TP_0843), HtpG (heat shock protein 90, locus tag: TP_0984), and DnaJ (locus tag: TP_0098). Comprehensive analyses were conducted to predict their antigenicity, solubility, virulence, signal peptide presence, physicochemical properties, and three-dimensional structures. Following the submission of the amino acid sequence to the ABCpred, IEDB MHC I Binding, and IEDB MHC II Binding servers, the B cell, CTL, and HTL epitopes of the vaccine construct were predicted. To develop an effective vaccine, a rigorous screening process was implemented to ensure the selection of epitopes that were antigenic, non-allergenic, and non-toxic. Among numerous B-cell epitopes, we preferentially selected those with high antigenicity and elevated ABC scores, which have been demonstrated to be effective in vaccines constructed in the study by Zhang et al. (2023). A new score was added for CTL and HTL epitopes each compared to the prediction of the properties of B-cell epitopes. The assessment included an analysis of the capacity of specific epitopes to induce interferon-gamma production by HTL epitopes, as well as the immunogenicity of CTL epitopes. On the basis of positive values, HTL epitopes with higher IFN scores and CTL epitopes with higher immunogenicity scores were selected to construct vaccines. Epitopes exhibiting these characteristics were prioritized due to their potential to provoke strong immune responses, which are crucial for the identification of infected host cells. Moreover, predicting T-cell epitopes derived from B cells is also a common approach for constructing multi-epitope vaccines. This study similarly predicted these epitopes as a reference, with detailed information provided in the Supplementary materials.

The ultimately developed vaccine comprised four B-cell epitopes, seven CTL epitopes, five HTL epitopes, and linkers including KK, AAY, and GPGPG. Subunit vaccines often face challenges in eliciting a robust immune response due to the restricted number of epitopes they display. However, the inclusion of adjuvants can substantially augment their immunostimulatory potential. Adjuvants enhance the immune response to vaccine antigens by engaging with the innate immune system (Verma et al., 2023). In this regard, *β*-defensin has been utilized as an adjuvant, in conjunction with an EAAAK linker at the N-terminus of the complex, to strategically enhance the immunological efficacy of subunit vaccines (Park et al., 2018). The vaccine also demonstrated high antigenicity, as evidenced by a Vaxijen score of 1.0843.

The two-dimensional structure of the vaccine revealed a composition of 34.66% alpha helices, 8.96% extended strands, 4.58% beta turns, and 51.79% random coils. The alpha helix primarily contributes stability and rigidity to the protein structure. In the analysis of physicochemical properties, the instability index and aliphatic index both suggest that the vaccine protein is likely to exhibit high stability and thermostability. Beta turns and random coils contribute significantly to the effective construction of the protein’s three-dimensional architecture. Some studies have also indicated that β-turns and random coil regions serve as primary recognition sites for leukocytes and antibodies, suggesting the vaccine’s potential for strong immunogenicity (Zhang et al., 2023). The quality of in-line protein tertiary structure construction frequently exhibits detailed imperfections. After optimizing the structure, to verify its tertiary quality, an Ramachandran plot provides an intuitive visualization of structural defects. Most of the residues were located in favored and allowed regions, suggesting that they formed tertiary structures with desirable properties. Furthermore, the z-score of −4.55 further corroborates the exceptional overall quality of the MSMV structure.

In this study, we designed a multi-epitope vaccine targeting the heat shock protein of *T. pallidum* through a reverse vaccinology approach. After constructing the finally designed vaccine, the vaccine was docked with three toll-like receptors and subsequent molecular dynamics simulations were performed on these vaccine-TLR complexes. Subsequently, the vaccine sequence was successfully cloned into the pET28a(+) vector to facilitate efficient expression and purification. An in *silico* evaluation of the vaccine’s immunogenicity was performed using the reputable C-ImmSim server, yielding valuable predictions concerning the elicited immune response. Although the bioinformatics results are valid, a comprehensive understanding of the vaccine’s efficacy and safety can only be achieved through a series of rigorous *in vitro* investigations.

### 4.2 Limitation

Bioinformatics analyses often primarily focus on antigens that are highly expressed or contain characteristic signaling peptides. As a result, certain low-abundance resistance factors that contribute to the immune response may be overlooked because the number of epitopes is limited. Furthermore, the linkers used in this study must undergo experimental validation to ensure they do not compromise the immunogenicity of the epitope or the overall safety of the vaccine. Ultimately, additional animal studies and clinical trials are necessary to confirm the practical efficacy of the vaccine combat *T. pallidum*.

## 5 Conclusion

*Treponema pallidum*, a sexually transmitted pathogen, is frequently misdiagnosed, particularly among men who have sex with men and are co-infected with HIV, posing a significant threat to global health. Previous studies have indicated that individuals with late latent syphilis demonstrate resistance to symptomatic reinfection by heterologous strains of *T. pallidum*. Additionally, repeated inoculation with gamma-irradiated *T. pallidum* has been shown to induce protective immunity in rabbits. These findings suggest that the development of protective vaccines is a viable possibility. In the present study, we present the development of an innovative multi-epitope vaccine designed to target heat shock proteins in *T. pallidum*. Through the systematic screening of B and T cell epitopes within the pathogen’s proteins, we engineered a vaccine characterized by its safety, non-allergenic nature, and enhanced antigenicity and specificity. Comprehensive evaluations, including protein structure assessment, immunoinformatics analysis, physicochemical prediction, and molecular dynamics simulations, confirmed that our vaccine possesses appropriate structural, physicochemical, and immunological properties. Following the application of reverse translation and codon optimization strategies, the ultimate vaccine construct was successfully cloned into the *E. coli* pET28a + plasmid, facilitating its effective expression and stability. It is crucial to emphasize that this reverse vaccine design requires comprehensive laboratory assessment to confirm its safety profile and immunological efficacy. The development of this vaccine holds the potential to serve as a crucial foundation for initiatives aimed at curbing the transmission of *T. pallidum*, thereby representing a substantial advancement in mitigating its prevalence and impact on public health.

## Data Availability

The datasets presented in this study can be found in online repositories. The names of the repository/repositories and accession number(s) can be found in the article/Supplementary material.
