# Supplementary material for: A comprehensive strategy for the development of a multi-epitope vaccine targeting Treponema pallidum, utilizing heat shock proteins, encompassing the entire process from vaccine design to in vitro evaluation of immunogenicity
Source: Front Microbiol. 2025 Mar 19;16:1551437. doi: 10.3389/fmicb.2025.1551437 (PMC11962626; doi:10.3389/fmicb.2025.1551437)
Supplement: Supplementary file 1 [file Data_Sheet_1.docx]

Supplementary Material

**Supplementary Table1.** The B-cell derived CTL epitopes of heat shock proteins.

| Number | Protein | Position | Peptide | Antigenicity | MHCPred HLA A*1101  IC50 Value (nM) |
| --- | --- | --- | --- | --- | --- |
| 1 | groEL | 384-392 | TEVEMKEKK | 1.7018 | 21.68 |
| 2 | GrpE | 36-44 | GEHSQELET | 1.6085 | 64.12 |
| 3 | dnaK | 222-230 | GGDDFDARI | 2.0577 | 52.84 |
| 4 | dnaK | 218-228 | DTHLGGDDFDA | 1.2678 | 48.31 |
| 5 | dnaK | 351-359 | FGKEGSKGV | 1.3149 | 55.72 |
| 6 | DnaJ | 183-192 | ATLLFSIWLL | 2.0560 | 16.07 |
| 7 | DnaJ | 167-175 | APSHSGSGA | 2.0439 | 26.61 |

**Supplementary Table 2.** The B-cell derived HTL epitopes of heat shock proteins.

| Number | Protein | Position | Peptide | Antigenicity | MHCPred DRB1*0101  IC50 Value (nM) |
| --- | --- | --- | --- | --- | --- |
| 1 | groEL | 387-395 | EMKEKKHRV | 1.3739 | 12.47 |
| 2 | dnaK | 351-359 | FGKEGSKGV | 1.3149 | 88.92 |
| 3 | DnaJ | 181-189 | IRATLLFSI | 1.0350 | 19.32 |

**Supplementary Table 3.**The websites used in this research.

| **Server Name** | **Website Address** |
| --- | --- |
| Vaxijen 2.0 | https://www.ddg-pharmfac.net/vaxijen |
| Protein-Sol | https://www.protein-sol.manchester.ac.uk |
| Virulentpred | https://bioinfo.icgeb.res.in/virulent/ |
| AlphaFold | https://golgi.sandbox.google.com/fold/ |
| ABCpred | http://www.webs.iiitd.edu.in/raghava/abcpred/ |
| AllerTOP v2.0 | https://www.ddg-pharmfac.net/AllerTOP/ |
| ToxinPred | http://crdd.osdd.net/raghava/toxinpred |
| IEDB MHC I | http://tools.iedb.org/mhci/ |
| IEDB MHC II | http://tools.iedb.org/mhcii/ |
| Class I Immunogenicity | http://tools.iedb.org/immunogenicity/ |
| IFNepitope | <http://crdd.osdd.net/raghava/ifnepitope/> |
| IEDB population coverage | http://tools.iedb.org/population/ |
| AggreProt | https://loschmidt.chemi.muni.cz/aggreprot/ |
| PSIPREDV3.3 | http://bioinf.cs.ucl.ac.uk/psipred/ |
| SOPMA | https://npsa.lyon.inserm.fr/cgi-bin/npsa_automat.pl?page=/NPSA/npsa_sopma.html |
| GalaxyRefine | https://galaxy.seoklab.org/cgi-bin/submit.cgi?type=REFINE |
| ProSA-web | https://prosa.services.came.sbg.ac.at/prosa.php |
| PROCHECK | https://saves.mbi.ucla.edu/ |
| ERRAT | https://saves.mbi.ucla.edu/ |
| PyMOL | https://pymol.org/ |
| ElliPro | http://tools.iedb.org/ellipro |
| Optimizer | https://genomes.urv.es/OPTIMIZER/ |
| SnapGene | https://www.snapgene.com/ |
| C-ImmSim | https://kraken.iac.rm.cnr.it/C-IMMSIM/ |
| Protein Data Bank | http://www.rcsb.org/ |
| HawkDock | http://cadd.zju.edu.cn/hawkdock/ |
| PDBsum | https://www.ebi.ac.uk/thornton-srv/databases/pdbsum/ |
| iMODS | https//imods.iqfr.csic.es/ |
| RNAfold | https://rna.tbi.univie.ac.at//cgi-bin/RNAWebSuite/RNAfold.cgi |

**Supplementary 4**

**Vaccines constructed by selected B cell and T cell epitopes.**

**20 vaccines randomly arranged by epitopes:**

**RV1**

GIINTLQKYYCRVRGGRCAVLSCLPKEEQIGKCSTRGRKCCRRKKEAAAKDLGLKLESADIALLGGPGPGADSLRASDPVPVESGGPGPGKREKIIAVYDLGGGTGPGPGEWELRAINRLGSEEEGPGPGQKEYDKDGAVTDTQKGPGPGTSEGQNAYTLDEVDAGPGPGNAAYAVLSDRASRARGPGPGAEDVEGEALAAYLESADIALLAAYYYPVLGERFAAYETGASEETLRAAYSAKDLGTGKAAYFVLAEELHVAAYKFDLLYARFAAYRTGDRRRARAAYAQYEFQTEVAAYLLFSIWLLRAAYLSDRASRARYAAYSGEGSVPGEHSQELETKKGDTHLGGDDFDARIVQKKHVIKDVFGKEGSKGVNKKYERTGDRRRARGYAQKKKRGRNGKSCVYCARAHAKKLGVSHRASTPEIKCAFKKHYLQKEYDKDGAVTDTKKPGSIRATLLFSIWLLRKKQERRGAPSHSGSGARPKKGGSYRAHGLQERRGAP

**RV2**

GIINTLQKYYCRVRGGRCAVLSCLPKEEQIGKCSTRGRKCCRRKKEAAAKADSLRASDPVPVESGGPGPGQKEYDKDGAVTDTQKGPGPGDLGLKLESADIALLGGPGPGEWELRAINRLGSEEEGPGPGKREKIIAVYDLGGGTGPGPGTSEGQNAYTLDEVDAGPGPGNAAYAVLSDRASRARGPGPGFVLAEELHVAAYLESADIALLAAYLSDRASRARYAAYAQYEFQTEVAAYAEDVEGEALAAYSAKDLGTGKAAYLLFSIWLLRAAYYYPVLGERFAAYKFDLLYARFAAYETGASEETLRAAYRTGDRRRARAAYHYLQKEYDKDGAVTDTKKHVIKDVFGKEGSKGVNKKGGSYRAHGLQERRGAPKKGDTHLGGDDFDARIVQKKRGRNGKSCVYCARAHAKKQERRGAPSHSGSGARPKKSGEGSVPGEHSQELETKKLGVSHRASTPEIKCAFKKYERTGDRRRARGYAQKKKPGSIRATLLFSIWLLR

**RV3**

GIINTLQKYYCRVRGGRCAVLSCLPKEEQIGKCSTRGRKCCRRKKEAAAKTSEGQNAYTLDEVDAGPGPGKREKIIAVYDLGGGTGPGPGEWELRAINRLGSEEEGPGPGADSLRASDPVPVESGGPGPGDLGLKLESADIALLGGPGPGNAAYAVLSDRASRARGPGPGQKEYDKDGAVTDTQKGPGPGLLFSIWLLRAAYKFDLLYARFAAYYYPVLGERFAAYFVLAEELHVAAYLESADIALLAAYAQYEFQTEVAAYLSDRASRARYAAYSAKDLGTGKAAYETGASEETLRAAYAEDVEGEALAAYRTGDRRRARAAYYERTGDRRRARGYAQKKKQERRGAPSHSGSGARPKKLGVSHRASTPEIKCAFKKSGEGSVPGEHSQELETKKPGSIRATLLFSIWLLRKKGGSYRAHGLQERRGAPKKHVIKDVFGKEGSKGVNKKGDTHLGGDDFDARIVQKKHYLQKEYDKDGAVTDTKKRGRNGKSCVYCARAHA

**RV4**

GIINTLQKYYCRVRGGRCAVLSCLPKEEQIGKCSTRGRKCCRRKKEAAAKNAAYAVLSDRASRARGPGPGDLGLKLESADIALLGGPGPGTSEGQNAYTLDEVDAGPGPGQKEYDKDGAVTDTQKGPGPGADSLRASDPVPVESGGPGPGEWELRAINRLGSEEEGPGPGKREKIIAVYDLGGGTGPGPGETGASEETLRAAYAQYEFQTEVAAYAEDVEGEALAAYLLFSIWLLRAAYKFDLLYARFAAYRTGDRRRARAAYLESADIALLAAYFVLAEELHVAAYSAKDLGTGKAAYLSDRASRARYAAYYYPVLGERFAAYGGSYRAHGLQERRGAPKKRGRNGKSCVYCARAHAKKGDTHLGGDDFDARIVQKKHYLQKEYDKDGAVTDTKKYERTGDRRRARGYAQKKKLGVSHRASTPEIKCAFKKQERRGAPSHSGSGARPKKPGSIRATLLFSIWLLRKKSGEGSVPGEHSQELETKKHVIKDVFGKEGSKGVN

**RV5**

GIINTLQKYYCRVRGGRCAVLSCLPKEEQIGKCSTRGRKCCRRKKEAAAKEWELRAINRLGSEEEGPGPGADSLRASDPVPVESGGPGPGKREKIIAVYDLGGGTGPGPGTSEGQNAYTLDEVDAGPGPGNAAYAVLSDRASRARGPGPGDLGLKLESADIALLGGPGPGQKEYDKDGAVTDTQKGPGPGRTGDRRRARAAYSAKDLGTGKAAYLSDRASRARYAAYYYPVLGERFAAYAQYEFQTEVAAYLESADIALLAAYKFDLLYARFAAYAEDVEGEALAAYLLFSIWLLRAAYFVLAEELHVAAYETGASEETLRAAYLGVSHRASTPEIKCAFKKSGEGSVPGEHSQELETKKPGSIRATLLFSIWLLRKKHVIKDVFGKEGSKGVNKKQERRGAPSHSGSGARPKKRGRNGKSCVYCARAHAKKGGSYRAHGLQERRGAPKKYERTGDRRRARGYAQKKKGDTHLGGDDFDARIVQKKHYLQKEYDKDGAVTDT

**RV6**

GIINTLQKYYCRVRGGRCAVLSCLPKEEQIGKCSTRGRKCCRRKKEAAAKDLGLKLESADIALLGGPGPGNAAYAVLSDRASRARGPGPGQKEYDKDGAVTDTQKGPGPGKREKIIAVYDLGGGTGPGPGEWELRAINRLGSEEEGPGPGADSLRASDPVPVESGGPGPGTSEGQNAYTLDEVDAGPGPGLESADIALLAAYLLFSIWLLRAAYFVLAEELHVAAYETGASEETLRAAYRTGDRRRARAAYLSDRASRARYAAYSAKDLGTGKAAYAQYEFQTEVAAYYYPVLGERFAAYKFDLLYARFAAYAEDVEGEALAAYGDTHLGGDDFDARIVQKKPGSIRATLLFSIWLLRKKQERRGAPSHSGSGARPKKYERTGDRRRARGYAQKKKHYLQKEYDKDGAVTDTKKSGEGSVPGEHSQELETKKRGRNGKSCVYCARAHAKKGGSYRAHGLQERRGAPKKHVIKDVFGKEGSKGVNKKLGVSHRASTPEIKCAF

**RV7**

GIINTLQKYYCRVRGGRCAVLSCLPKEEQIGKCSTRGRKCCRRKKEAAAKQKEYDKDGAVTDTQKGPGPGTSEGQNAYTLDEVDAGPGPGADSLRASDPVPVESGGPGPGDLGLKLESADIALLGGPGPGNAAYAVLSDRASRARGPGPGKREKIIAVYDLGGGTGPGPGEWELRAINRLGSEEEGPGPGLSDRASRARYAAYYYPVLGERFAAYKFDLLYARFAAYAEDVEGEALAAYFVLAEELHVAAYETGASEETLRAAYAQYEFQTEVAAYLLFSIWLLRAAYLESADIALLAAYRTGDRRRARAAYSAKDLGTGKAAYQERRGAPSHSGSGARPKKHYLQKEYDKDGAVTDTKKHVIKDVFGKEGSKGVNKKRGRNGKSCVYCARAHAKKLGVSHRASTPEIKCAFKKGDTHLGGDDFDARIVQKKPGSIRATLLFSIWLLRKKSGEGSVPGEHSQELETKKGGSYRAHGLQERRGAPKKYERTGDRRRARGYAQK

**RV8**

GIINTLQKYYCRVRGGRCAVLSCLPKEEQIGKCSTRGRKCCRRKKEAAAKKREKIIAVYDLGGGTGPGPGEWELRAINRLGSEEEGPGPGNAAYAVLSDRASRARGPGPGADSLRASDPVPVESGGPGPGTSEGQNAYTLDEVDAGPGPGQKEYDKDGAVTDTQKGPGPGDLGLKLESADIALLGGPGPGSAKDLGTGKAAYRTGDRRRARAAYLESADIALLAAYKFDLLYARFAAYLSDRASRARYAAYAEDVEGEALAAYYYPVLGERFAAYETGASEETLRAAYAQYEFQTEVAAYLLFSIWLLRAAYFVLAEELHVAAYRGRNGKSCVYCARAHAKKGGSYRAHGLQERRGAPKKYERTGDRRRARGYAQKKKLGVSHRASTPEIKCAFKKSGEGSVPGEHSQELETKKHVIKDVFGKEGSKGVNKKHYLQKEYDKDGAVTDTKKQERRGAPSHSGSGARPKKPGSIRATLLFSIWLLRKKGDTHLGGDDFDARIVQ

**RV9**

GIINTLQKYYCRVRGGRCAVLSCLPKEEQIGKCSTRGRKCCRRKKEAAAKADSLRASDPVPVESGGPGPGDLGLKLESADIALLGGPGPGTSEGQNAYTLDEVDAGPGPGEWELRAINRLGSEEEGPGPGQKEYDKDGAVTDTQKGPGPGKREKIIAVYDLGGGTGPGPGNAAYAVLSDRASRARGPGPGAQYEFQTEVAAYAEDVEGEALAAYETGASEETLRAAYSAKDLGTGKAAYLLFSIWLLRAAYFVLAEELHVAAYRTGDRRRARAAYLESADIALLAAYLSDRASRARYAAYYYPVLGERFAAYKFDLLYARFAAYHVIKDVFGKEGSKGVNKKLGVSHRASTPEIKCAFKKSGEGSVPGEHSQELETKKGGSYRAHGLQERRGAPKKGDTHLGGDDFDARIVQKKYERTGDRRRARGYAQKKKPGSIRATLLFSIWLLRKKRGRNGKSCVYCARAHAKKHYLQKEYDKDGAVTDTKKQERRGAPSHSGSGARP

**RV10**

GIINTLQKYYCRVRGGRCAVLSCLPKEEQIGKCSTRGRKCCRRKKEAAAKNAAYAVLSDRASRARGPGPGQKEYDKDGAVTDTQKGPGPGEWELRAINRLGSEEEGPGPGDLGLKLESADIALLGGPGPGKREKIIAVYDLGGGTGPGPGADSLRASDPVPVESGGPGPGTSEGQNAYTLDEVDAGPGPGYYPVLGERFAAYFVLAEELHVAAYLLFSIWLLRAAYRTGDRRRARAAYETGASEETLRAAYKFDLLYARFAAYAEDVEGEALAAYLSDRASRARYAAYSAKDLGTGKAAYLESADIALLAAYAQYEFQTEVAAYPGSIRATLLFSIWLLRKKGDTHLGGDDFDARIVQKKHYLQKEYDKDGAVTDTKKQERRGAPSHSGSGARPKKHVIKDVFGKEGSKGVNKKGGSYRAHGLQERRGAPKKLGVSHRASTPEIKCAFKKYERTGDRRRARGYAQKKKRGRNGKSCVYCARAHAKKSGEGSVPGEHSQELET

**RV11**

GIINTLQKYYCRVRGGRCAVLSCLPKEEQIGKCSTRGRKCCRRKKEAAAKTSEGQNAYTLDEVDAGPGPGNAAYAVLSDRASRARGPGPGDLGLKLESADIALLGGPGPGQKEYDKDGAVTDTQKGPGPGEWELRAINRLGSEEEGPGPGADSLRASDPVPVESGGPGPGKREKIIAVYDLGGGTGPGPGKFDLLYARFAAYLSDRASRARYAAYSAKDLGTGKAAYLESADIALLAAYYYPVLGERFAAYLLFSIWLLRAAYETGASEETLRAAYRTGDRRRARAAYFVLAEELHVAAYAQYEFQTEVAAYAEDVEGEALAAYSGEGSVPGEHSQELETKKYERTGDRRRARGYAQKKKRGRNGKSCVYCARAHAKKPGSIRATLLFSIWLLRKKGGSYRAHGLQERRGAPKKHYLQKEYDKDGAVTDTKKGDTHLGGDDFDARIVQKKHVIKDVFGKEGSKGVNKKQERRGAPSHSGSGARPKKLGVSHRASTPEIKCAF

**RV12**

GIINTLQKYYCRVRGGRCAVLSCLPKEEQIGKCSTRGRKCCRRKKEAAAKKREKIIAVYDLGGGTGPGPGADSLRASDPVPVESGGPGPGQKEYDKDGAVTDTQKGPGPGNAAYAVLSDRASRARGPGPGTSEGQNAYTLDEVDAGPGPGDLGLKLESADIALLGGPGPGEWELRAINRLGSEEEGPGPGAEDVEGEALAAYETGASEETLRAAYAQYEFQTEVAAYLSDRASRARYAAYSAKDLGTGKAAYYYPVLGERFAAYFVLAEELHVAAYKFDLLYARFAAYRTGDRRRARAAYLLFSIWLLRAAYLESADIALLAAYGGSYRAHGLQERRGAPKKHVIKDVFGKEGSKGVNKKLGVSHRASTPEIKCAFKKQERRGAPSHSGSGARPKKGDTHLGGDDFDARIVQKKSGEGSVPGEHSQELETKKYERTGDRRRARGYAQKKKHYLQKEYDKDGAVTDTKKRGRNGKSCVYCARAHAKKPGSIRATLLFSIWLLR

**RV13**

GIINTLQKYYCRVRGGRCAVLSCLPKEEQIGKCSTRGRKCCRRKKEAAAKEWELRAINRLGSEEEGPGPGDLGLKLESADIALLGGPGPGADSLRASDPVPVESGGPGPGKREKIIAVYDLGGGTGPGPGQKEYDKDGAVTDTQKGPGPGTSEGQNAYTLDEVDAGPGPGNAAYAVLSDRASRARGPGPGLLFSIWLLRAAYLESADIALLAAYRTGDRRRARAAYFVLAEELHVAAYAQYEFQTEVAAYSAKDLGTGKAAYLSDRASRARYAAYAEDVEGEALAAYETGASEETLRAAYKFDLLYARFAAYYYPVLGERFAAYHYLQKEYDKDGAVTDTKKRGRNGKSCVYCARAHAKKPGSIRATLLFSIWLLRKKGDTHLGGDDFDARIVQKKLGVSHRASTPEIKCAFKKYERTGDRRRARGYAQKKKGGSYRAHGLQERRGAPKKQERRGAPSHSGSGARPKKSGEGSVPGEHSQELETKKHVIKDVFGKEGSKGVN

**RV14**

GIINTLQKYYCRVRGGRCAVLSCLPKEEQIGKCSTRGRKCCRRKKEAAAKQKEYDKDGAVTDTQKGPGPGKREKIIAVYDLGGGTGPGPGTSEGQNAYTLDEVDAGPGPGADSLRASDPVPVESGGPGPGNAAYAVLSDRASRARGPGPGEWELRAINRLGSEEEGPGPGDLGLKLESADIALLGGPGPGFVLAEELHVAAYKFDLLYARFAAYYYPVLGERFAAYLLFSIWLLRAAYLESADIALLAAYRTGDRRRARAAYETGASEETLRAAYAQYEFQTEVAAYAEDVEGEALAAYSAKDLGTGKAAYLSDRASRARYAAYYERTGDRRRARGYAQKKKSGEGSVPGEHSQELETKKQERRGAPSHSGSGARPKKHYLQKEYDKDGAVTDTKKHVIKDVFGKEGSKGVNKKPGSIRATLLFSIWLLRKKRGRNGKSCVYCARAHAKKGGSYRAHGLQERRGAPKKGDTHLGGDDFDARIVQKKLGVSHRASTPEIKCAF

**RV15**

GIINTLQKYYCRVRGGRCAVLSCLPKEEQIGKCSTRGRKCCRRKKEAAAKDLGLKLESADIALLGGPGPGEWELRAINRLGSEEEGPGPGNAAYAVLSDRASRARGPGPGTSEGQNAYTLDEVDAGPGPGADSLRASDPVPVESGGPGPGQKEYDKDGAVTDTQKGPGPGKREKIIAVYDLGGGTGPGPGETGASEETLRAAYLSDRASRARYAAYAEDVEGEALAAYAQYEFQTEVAAYKFDLLYARFAAYFVLAEELHVAAYLLFSIWLLRAAYYYPVLGERFAAYLESADIALLAAYRTGDRRRARAAYSAKDLGTGKAAYLGVSHRASTPEIKCAFKKGGSYRAHGLQERRGAPKKGDTHLGGDDFDARIVQKKRGRNGKSCVYCARAHAKKQERRGAPSHSGSGARPKKHVIKDVFGKEGSKGVNKKSGEGSVPGEHSQELETKKPGSIRATLLFSIWLLRKKYERTGDRRRARGYAQKKKHYLQKEYDKDGAVTDT

**RV16**

GIINTLQKYYCRVRGGRCAVLSCLPKEEQIGKCSTRGRKCCRRKKEAAAKADSLRASDPVPVESGGPGPGTSEGQNAYTLDEVDAGPGPGKREKIIAVYDLGGGTGPGPGNAAYAVLSDRASRARGPGPGDLGLKLESADIALLGGPGPGEWELRAINRLGSEEEGPGPGQKEYDKDGAVTDTQKGPGPGRTGDRRRARAAYYYPVLGERFAAYSAKDLGTGKAAYETGASEETLRAAYLSDRASRARYAAYLESADIALLAAYKFDLLYARFAAYLLFSIWLLRAAYFVLAEELHVAAYAEDVEGEALAAYAQYEFQTEVAAYHVIKDVFGKEGSKGVNKKHYLQKEYDKDGAVTDTKKYERTGDRRRARGYAQKKKSGEGSVPGEHSQELETKKGGSYRAHGLQERRGAPKKLGVSHRASTPEIKCAFKKQERRGAPSHSGSGARPKKGDTHLGGDDFDARIVQKKPGSIRATLLFSIWLLRKKRGRNGKSCVYCARAHA

**RV17**

GIINTLQKYYCRVRGGRCAVLSCLPKEEQIGKCSTRGRKCCRRKKEAAAKNAAYAVLSDRASRARGPGPGKREKIIAVYDLGGGTGPGPGEWELRAINRLGSEEEGPGPGQKEYDKDGAVTDTQKGPGPGTSEGQNAYTLDEVDAGPGPGDLGLKLESADIALLGGPGPGADSLRASDPVPVESGGPGPGLESADIALLAAYAQYEFQTEVAAYFVLAEELHVAAYAEDVEGEALAAYLLFSIWLLRAAYETGASEETLRAAYRTGDRRRARAAYSAKDLGTGKAAYLSDRASRARYAAYYYPVLGERFAAYKFDLLYARFAAYQERRGAPSHSGSGARPKKPGSIRATLLFSIWLLRKKSGEGSVPGEHSQELETKKLGVSHRASTPEIKCAFKKRGRNGKSCVYCARAHAKKGDTHLGGDDFDARIVQKKHYLQKEYDKDGAVTDTKKHVIKDVFGKEGSKGVNKKGGSYRAHGLQERRGAPKKYERTGDRRRARGYAQK

**RV18**

GIINTLQKYYCRVRGGRCAVLSCLPKEEQIGKCSTRGRKCCRRKKEAAAKTSEGQNAYTLDEVDAGPGPGDLGLKLESADIALLGGPGPGQKEYDKDGAVTDTQKGPGPGDLGLKLESADIALLGGPGPGKREKIIAVYDLGGGTGPGPGNAAYAVLSDRASRARGPGPGEWELRAINRLGSEEEGPGPGLSDRASRARYAAYSAKDLGTGKAAYLLFSIWLLRAAYKFDLLYARFAAYFVLAEELHVAAYAQYEFQTEVAAYYYPVLGERFAAYLESADIALLAAYETGASEETLRAAYAEDVEGEALAAYRTGDRRRARAAYGDTHLGGDDFDARIVQKKLGVSHRASTPEIKCAFKKGGSYRAHGLQERRGAPKKYERTGDRRRARGYAQKKKPGSIRATLLFSIWLLRKKRGRNGKSCVYCARAHAKKHVIKDVFGKEGSKGVNKKSGEGSVPGEHSQELETKKHYLQKEYDKDGAVTDTKKQERRGAPSHSGSGARP

**RV19**

GIINTLQKYYCRVRGGRCAVLSCLPKEEQIGKCSTRGRKCCRRKKEAAAKKREKIIAVYDLGGGTGPGPGQKEYDKDGAVTDTQKGPGPGDLGLKLESADIALLGGPGPGEWELRAINRLGSEEEGPGPGADSLRASDPVPVESGGPGPGTSEGQNAYTLDEVDAGPGPGNAAYAVLSDRASRARGPGPGSAKDLGTGKAAYAEDVEGEALAAYETGASEETLRAAYRTGDRRRARAAYYYPVLGERFAAYLSDRASRARYAAYLESADIALLAAYFVLAEELHVAAYLLFSIWLLRAAYAQYEFQTEVAAYKFDLLYARFAAYRGRNGKSCVYCARAHAKKQERRGAPSHSGSGARPKKHYLQKEYDKDGAVTDTKKGGSYRAHGLQERRGAPKKSGEGSVPGEHSQELETKKYERTGDRRRARGYAQKKKLGVSHRASTPEIKCAFKKGDTHLGGDDFDARIVQKKHVIKDVFGKEGSKGVNKKPGSIRATLLFSIWLLR

**RV20**

GIINTLQKYYCRVRGGRCAVLSCLPKEEQIGKCSTRGRKCCRRKKEAAAKEWELRAINRLGSEEEGPGPGNAAYAVLSDRASRARGPGPGADSLRASDPVPVESGGPGPGTSEGQNAYTLDEVDAGPGPGDLGLKLESADIALLGGPGPGQKEYDKDGAVTDTQKGPGPGKREKIIAVYDLGGGTGPGPGAQYEFQTEVAAYLLFSIWLLRAAYLESADIALLAAYYYPVLGERFAAYAEDVEGEALAAYKFDLLYARFAAYSAKDLGTGKAAYETGASEETLRAAYRTGDRRRARAAYLSDRASRARYAAYFVLAEELHVAAYPGSIRATLLFSIWLLRKKHVIKDVFGKEGSKGVNKKGDTHLGGDDFDARIVQKKQERRGAPSHSGSGARPKKHYLQKEYDKDGAVTDTKKGGSYRAHGLQERRGAPKKYERTGDRRRARGYAQKKKRGRNGKSCVYCARAHAKKLGVSHRASTPEIKCAFKKSGEGSVPGEHSQELET

**Vaccine evaluation:**

**Properties prediction.**

| **Vaccine**  **number** | **Antigenicity** | **Instability index** | **Solubility** | **Aggregation** | **Disorder** | **Sequence identity (%)** |
| --- | --- | --- | --- | --- | --- | --- |
| RV1 | 1.0843 | 33.20 | 0.948 | -22.20 | 0.433 | 0% |
| RV2 | 1.0863 | 34.25 | 0.948 | -22.20 | 0.434 | 53.8% |
| RV3 | 1.0622 | 34.25 | 0.948 | -22.00 | 0.428 | 53.2% |
| RV4 | 1.0696 | 33.54 | 0.948 | -22.10 | 0.433 | 48.7% |
| RV5 | 1.1019 | 34.50 | 0.949 | -22.20 | 0.437 | 52.4% |
| RV6 | 1.0893 | 34.14 | 0.949 | -22.50 | 0.438 | 55.2% |
| RV7 | 1.0839 | 33.84 | 0.949 | -22.30 | 0.423 | 49.5% |
| RV8 | 1.0724 | 33.02 | 0.948 | -22.50 | 0.435 | 44.0% |
| RV9 | 1.0946 | 33.61 | 0.948 | -22.20 | 0.431 | 54.5% |
| RV10 | 1.0560 | 33.99 | 0.949 | -22.20 | 0.422 | 42.6% |
| RV11 | 1.0594 | 34.14 | 0.949 | -22.50 | 0.423 | 46.8% |
| RV12 | 1.0826 | 33.37 | 0.947 | -22.20 | 0.441 | 51.4% |
| RV13 | 1.0722 | 33.76 | 0.948 | -22.10 | 0.418 | 55.0% |
| RV14 | 1.0877 | 34.86 | 0.948 | -22.50 | 0.432 | 47.1% |
| RV15 | 1.0907 | 34.01 | 0.949 | -22.20 | 0.444 | 49.5% |
| RV16 | 1.0969 | 34.59 | 0.949 | -22.00 | 0.445 | 48.2% |
| RV17 | 1.0715 | 33.54 | 0.948 | -22.30 | 0.424 | 53.5% |
| RV18 | 1.0598 | 31.92 | 0.941 | -21.10 | 0.395 | 46.7% |
| RV19 | 1.1022 | 31.92 | 0.948 | -22.20 | 0.446 | 50.4% |
| RV20 | 1.0896 | 33.75 | 0.947 | -22.20 | 0.431 | 53.5% |

**After evaluation, it was found that there were minimal differences in the properties among the 20 vaccines. Consequently, the first five vaccines were selected for further analysis.**

**Five vaccines as following: RV1, RV2, RV3, RV4, RV5**

**Evaluation in the C-IMMSIM server:**

**
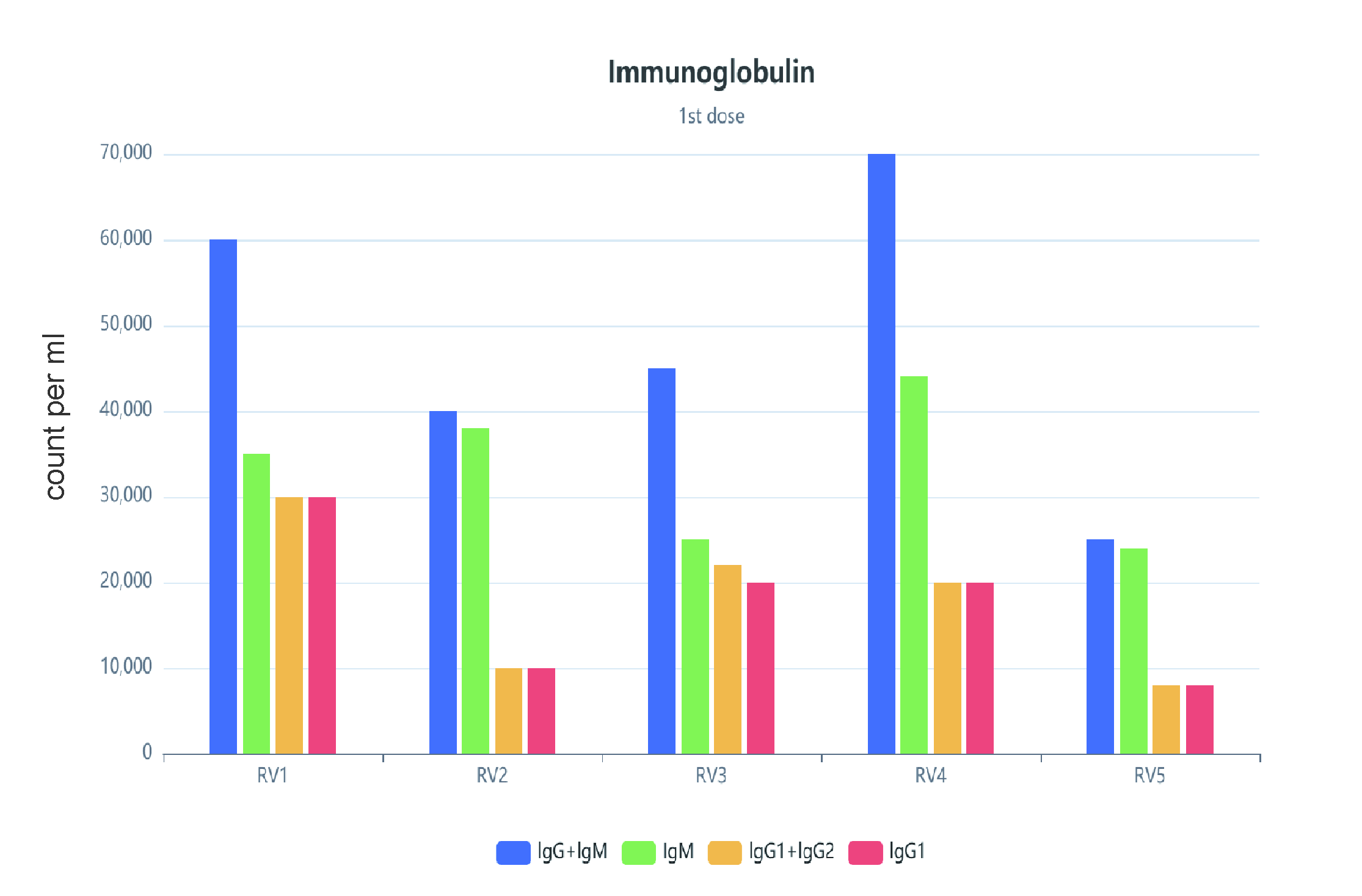
**

**
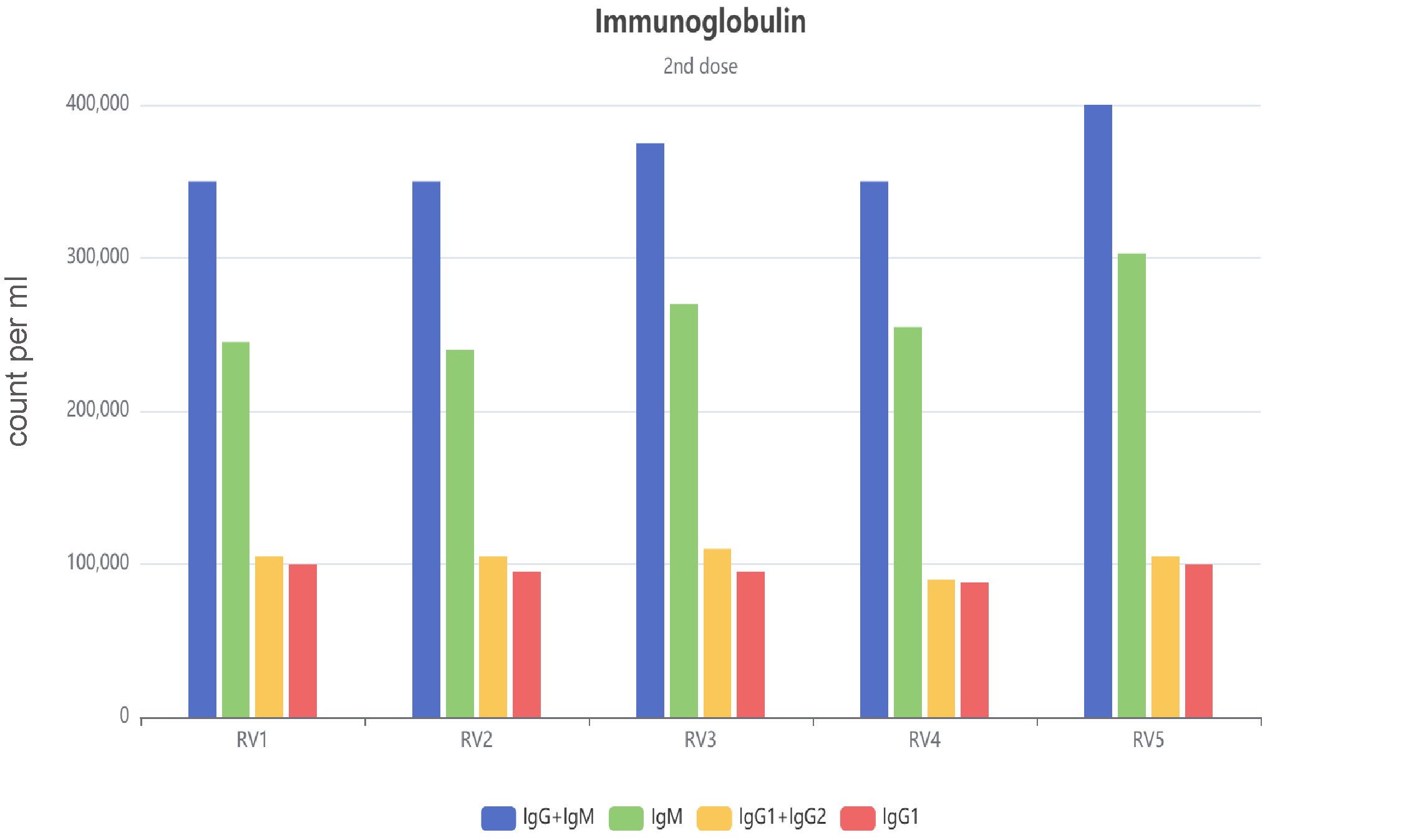
**

**
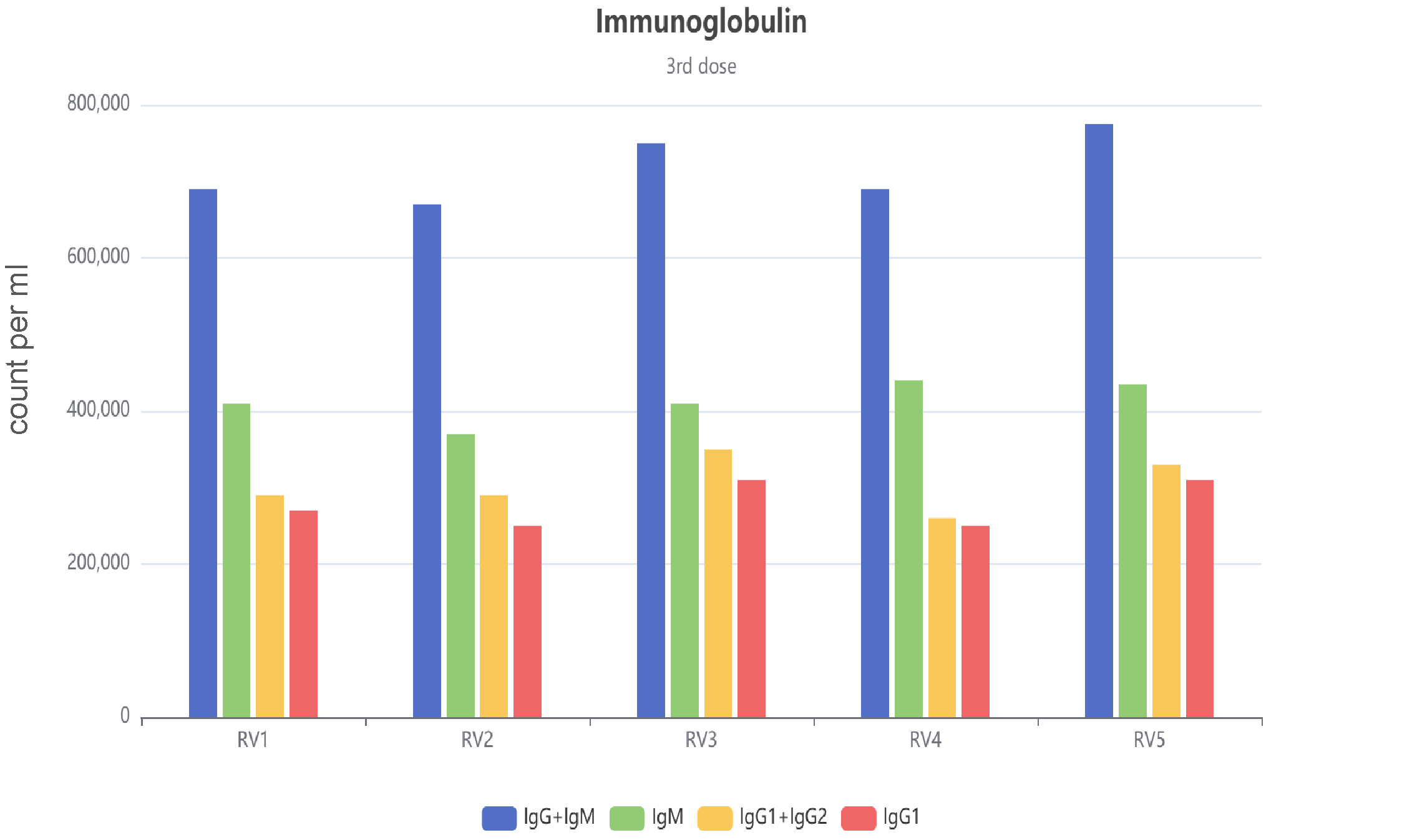
**

**
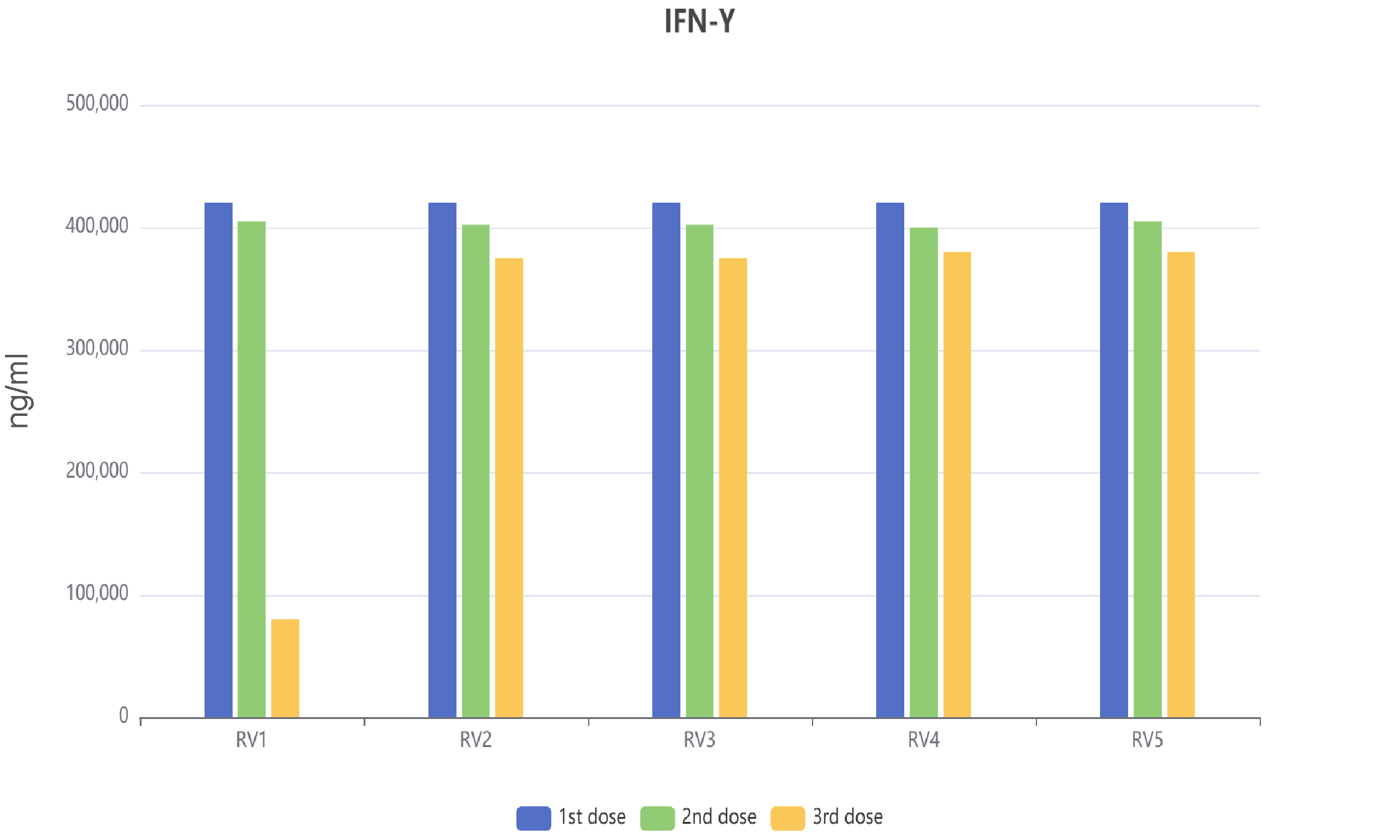
**

**From the above analysis, it is evident that the properties of the vaccine we constructed remained largely unchanged following the alteration in epitope ranking. Therefore, we continued to use the initial vaccine for subsequent analysis.**

**Supplementary 5**

**New vaccines constructed by B cell derived T cell epitopes and adjuvant.**

**20 vaccines randomly arranged by epitopes:**

**1**

**GIINTLQKYYCRVRGGRCAVLSCLPKEEQIGKCSTRGRKCCRRKKEAAAKGGDDFDARIAAYAPSHSGSGAAAYTEVEMKEKKAAYFGKEGSKGVAAYGEHSQELETAAYATLLFSIWLLAAYDTHLGGDDFDAAAYEMKEKKHRVGPGPGFGKEGSKGVGPGPGIRATLLFSI**

**2**

**GIINTLQKYYCRVRGGRCAVLSCLPKEEQIGKCSTRGRKCCRRKKEAAAKATLLFSIWLLAAYGEHSQELETAAYDTHLGGDDFDAAAYTEVEMKEKKAAYAPSHSGSGAAAYGGDDFDARIAAYFGKEGSKGVAAYEMKEKKHRVGPGPGIRATLLFSIGPGPGFGKEGSKGV**

**3**

**GIINTLQKYYCRVRGGRCAVLSCLPKEEQIGKCSTRGRKCCRRKKEAAAKFGKEGSKGVAAYTEVEMKEKKAAYGGDDFDARIAAYATLLFSIWLLAAYDTHLGGDDFDAAAYGEHSQELETAAYAPSHSGSGAAAYFGKEGSKGVGPGPGEMKEKKHRVGPGPGIRATLLFSI**

**4**

**GIINTLQKYYCRVRGGRCAVLSCLPKEEQIGKCSTRGRKCCRRKKEAAAKGEHSQELETAAYATLLFSIWLLAAYAPSHSGSGAAAYGGDDFDARIAAYFGKEGSKGVAAYTEVEMKEKKAAYDTHLGGDDFDAAAYFGKEGSKGVGPGPGIRATLLFSIGPGPGEMKEKKHRV**

**5**

**GIINTLQKYYCRVRGGRCAVLSCLPKEEQIGKCSTRGRKCCRRKKEAAAKDTHLGGDDFDAAAYFGKEGSKGVAAYGEHSQELETAAYAPSHSGSGAAAYTEVEMKEKKAAYATLLFSIWLLAAYGGDDFDARIAAYIRATLLFSIGPGPGEMKEKKHRVGPGPGFGKEGSKGV**

**6**

**GIINTLQKYYCRVRGGRCAVLSCLPKEEQIGKCSTRGRKCCRRKKEAAAKAPSHSGSGAAAYGGDDFDARIAAYATLLFSIWLLAAYGEHSQELETAAYDTHLGGDDFDAAAYFGKEGSKGVAAYTEVEMKEKKAAYIRATLLFSIGPGPGFGKEGSKGVGPGPGEMKEKKHRV**

**7**

**GIINTLQKYYCRVRGGRCAVLSCLPKEEQIGKCSTRGRKCCRRKKEAAAKTEVEMKEKKAAYDTHLGGDDFDAAAYFGKEGSKGVAAYGGDDFDARIAAYAPSHSGSGAAAYGEHSQELETAAYATLLFSIWLLAAYEMKEKKHRVGPGPGFGKEGSKGVGPGPGIRATLLFSI**

**8**

**GIINTLQKYYCRVRGGRCAVLSCLPKEEQIGKCSTRGRKCCRRKKEAAAKGGDDFDARIAAYGEHSQELETAAYATLLFSIWLLAAYAPSHSGSGAAAYTEVEMKEKKAAYFGKEGSKGVAAYDTHLGGDDFDAAAYEMKEKKHRVGPGPGIRATLLFSIGPGPGFGKEGSKGV**

**9**

**GIINTLQKYYCRVRGGRCAVLSCLPKEEQIGKCSTRGRKCCRRKKEAAAKFGKEGSKGVAAYAPSHSGSGAAAYDTHLGGDDFDAAAYGEHSQELETAAYATLLFSIWLLAAYTEVEMKEKKAAYGGDDFDARIAAYFGKEGSKGVGPGPGEMKEKKHRVGPGPGIRATLLFSI**

**10**

**GIINTLQKYYCRVRGGRCAVLSCLPKEEQIGKCSTRGRKCCRRKKEAAAKATLLFSIWLLAAYTEVEMKEKKAAYGEHSQELETAAYFGKEGSKGVAAYGGDDFDARIAAYAPSHSGSGAAAYDTHLGGDDFDAAAYFGKEGSKGVGPGPGIRATLLFSIGPGPGEMKEKKHRV**

**11**

**GIINTLQKYYCRVRGGRCAVLSCLPKEEQIGKCSTRGRKCCRRKKEAAAKGEHSQELETAAYGGDDFDARIAAYTEVEMKEKKAAYDTHLGGDDFDAAAYATLLFSIWLLAAYAPSHSGSGAAAYFGKEGSKGVAAYIRATLLFSIGPGPGEMKEKKHRVGPGPGFGKEGSKGV**

**12**

**GIINTLQKYYCRVRGGRCAVLSCLPKEEQIGKCSTRGRKCCRRKKEAAAKDTHLGGDDFDAAAYAPSHSGSGAAAYGGDDFDARIAAYTEVEMKEKKAAYFGKEGSKGVAAYATLLFSIWLLAAYGEHSQELETAAYIRATLLFSIGPGPGFGKEGSKGVGPGPGEMKEKKHRV**

**13**

**GIINTLQKYYCRVRGGRCAVLSCLPKEEQIGKCSTRGRKCCRRKKEAAAKAPSHSGSGAAAYFGKEGSKGVAAYTEVEMKEKKAAYATLLFSIWLLAAYGEHSQELETAAYDTHLGGDDFDAAAYGGDDFDARIAAYEMKEKKHRVGPGPGFGKEGSKGVGPGPGIRATLLFSI**

**14**

**GIINTLQKYYCRVRGGRCAVLSCLPKEEQIGKCSTRGRKCCRRKKEAAAKTEVEMKEKKAAYATLLFSIWLLAAYAPSHSGSGAAAYDTHLGGDDFDAAAYGGDDFDARIAAYFGKEGSKGVAAYGEHSQELETAAYEMKEKKHRVGPGPGIRATLLFSIGPGPGFGKEGSKGV**

**15**

**GIINTLQKYYCRVRGGRCAVLSCLPKEEQIGKCSTRGRKCCRRKKEAAAKGGDDFDARIAAYDTHLGGDDFDAAAYFGKEGSKGVAAYGEHSQELETAAYAPSHSGSGAAAYTEVEMKEKKAAYATLLFSIWLLAAYFGKEGSKGVGPGPGEMKEKKHRVGPGPGIRATLLFSI**

**16**

**GIINTLQKYYCRVRGGRCAVLSCLPKEEQIGKCSTRGRKCCRRKKEAAAKATLLFSIWLLAAYAPSHSGSGAAAYGEHSQELETAAYGGDDFDARIAAYTEVEMKEKKAAYDTHLGGDDFDAAAYFGKEGSKGVAAYFGKEGSKGVGPGPGIRATLLFSIGPGPGEMKEKKHRV**

**17**

**GIINTLQKYYCRVRGGRCAVLSCLPKEEQIGKCSTRGRKCCRRKKEAAAKGEHSQELETAAYFGKEGSKGVAAYATLLFSIWLLAAYTEVEMKEKKAAYDTHLGGDDFDAAAYGGDDFDARIAAYAPSHSGSGAAAYIRATLLFSIGPGPGEMKEKKHRVGPGPGFGKEGSKGV**

**18**

**GIINTLQKYYCRVRGGRCAVLSCLPKEEQIGKCSTRGRKCCRRKKEAAAKFGKEGSKGVAAYGGDDFDARIAAYAPSHSGSGAAAYDTHLGGDDFDAAAYTEVEMKEKKAAYGEHSQELETAAYATLLFSIWLLAAYIRATLLFSIGPGPGFGKEGSKGVGPGPGEMKEKKHRV**

**19**

**GIINTLQKYYCRVRGGRCAVLSCLPKEEQIGKCSTRGRKCCRRKKEAAAKDTHLGGDDFDAAAYTEVEMKEKKAAYATLLFSIWLLAAYFGKEGSKGVAAYGEHSQELETAAYAPSHSGSGAAAYGGDDFDARIAAYEMKEKKHRVGPGPGFGKEGSKGVGPGPGIRATLLFSI**

**20**

**GIINTLQKYYCRVRGGRCAVLSCLPKEEQIGKCSTRGRKCCRRKKEAAAKAPSHSGSGAAAYGEHSQELETAAYGGDDFDARIAAYATLLFSIWLLAAYFGKEGSKGVAAYTEVEMKEKKAAYDTHLGGDDFDAAAYEMKEKKHRVGPGPGIRATLLFSIGPGPGFGKEGSKGV**

**Vaccine evaluation:**

**Properties prediction.**

| **Vaccine**  **number** | **Antigenicity** | **Instability index** | **Solubility** | **Aggregation** | **Disorder** | **Sequence identity (%)** |
| --- | --- | --- | --- | --- | --- | --- |
| **1** | **1.1164** | **25.92** | **0.884** | **-14.70** | **0.272** | **0.0%** |
| **2** | **1.0061** | **25.05** | **0.841** | **-14.70** | **0.264** | **54.8%** |
| **3** | **1.0427** | **25.92** | **0.885** | **-14.70** | **0.277** | **64.6%** |
| **4** | **1.0441** | **26.41** | **0.870** | **-14.60** | **0.257** | **62.9%** |
| **5** | **1.0136** | **25.54** | **0.889** | **-14.70** | **0.270** | **67.8%** |
| **6** | **0.9969** | **25.54** | **0.889** | **-14.60** | **0.265** | **63.8%** |
| **7** | **1.0934** | **26.41** | **0.883** | **-14.70** | **0.271** | **70.0%** |
| **8** | **1.0962** | **26.41** | **0.888** | **-14.70** | **0.256** | **68.1%** |
| **9** | **1.0491** | **25.92** | **0.885** | **-14.70** | **0.269** | **70.7%** |
| **10** | **0.9753** | **25.05** | **0.804** | **-14.60** | **0.265** | **65.7%** |
| **11** | **1.0647** | **26.90** | **0.888** | **-14.70** | **0.266** | **62.0%** |
| **12** | **0.9964** | **25.54** | **0.889** | **-14.60** | **0.265** | **69.8%** |
| **13** | **1.0764** | **24.56** | **0.885** | **-14.70** | **0.267** | **76.8%** |
| **14** | **1.0962** | **26.90** | **0.860** | **-14.70** | **0.261** | **57.1%** |
| **15** | **1.0682** | **25.92** | **0.881** | **-14.70** | **0.279** | **65.8%** |
| **16** | **0.9978** | **25.05** | **0.797** | **-14.60** | **0.264** | **67.4%** |
| **17** | **1.0567** | **26.90** | **0.888** | **-14.70** | **0.266** | **60.6%** |
| **18** | **1.0437** | **26.90** | **0.889** | **-14.60** | **0.282** | **70.4%** |
| **19** | **1.1108** | **24.56** | **0.885** | **-14.70** | **0.271** | **78.5%** |
| **20** | **1.0916** | **25.05** | **0.888** | **-14.70** | **0.267** | **60.0%** |

**After evaluation, it was found that there were minimal differences in the properties among the 20 vaccines. Consequently, five vaccines with particularly favorable characteristics were selected for further analysis.**

**Five vaccines as following: Vaccine4( V4), V8,V9,V13,V20.**

**Evaluation in the C-IMMSIM server:**

**
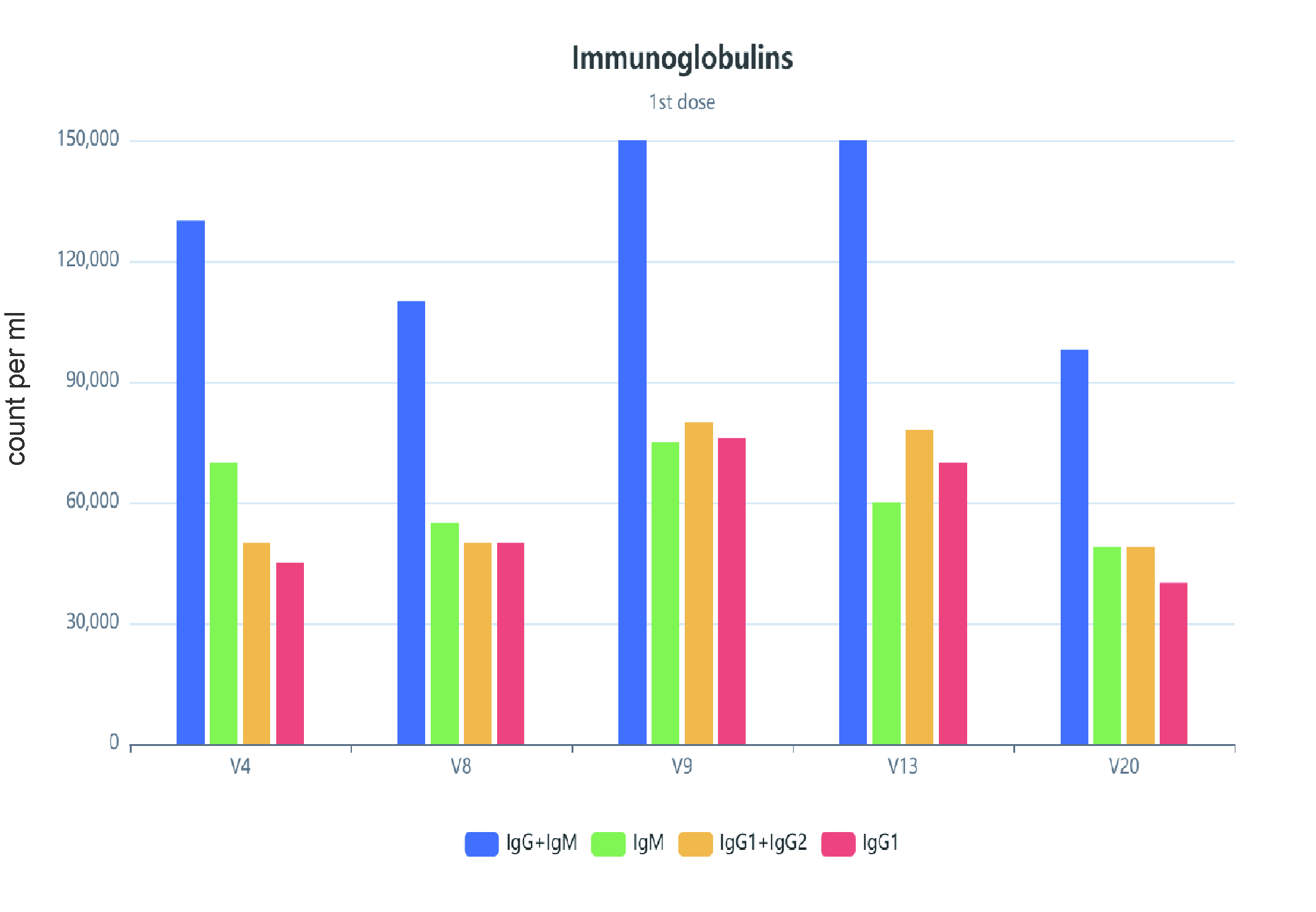
**

**
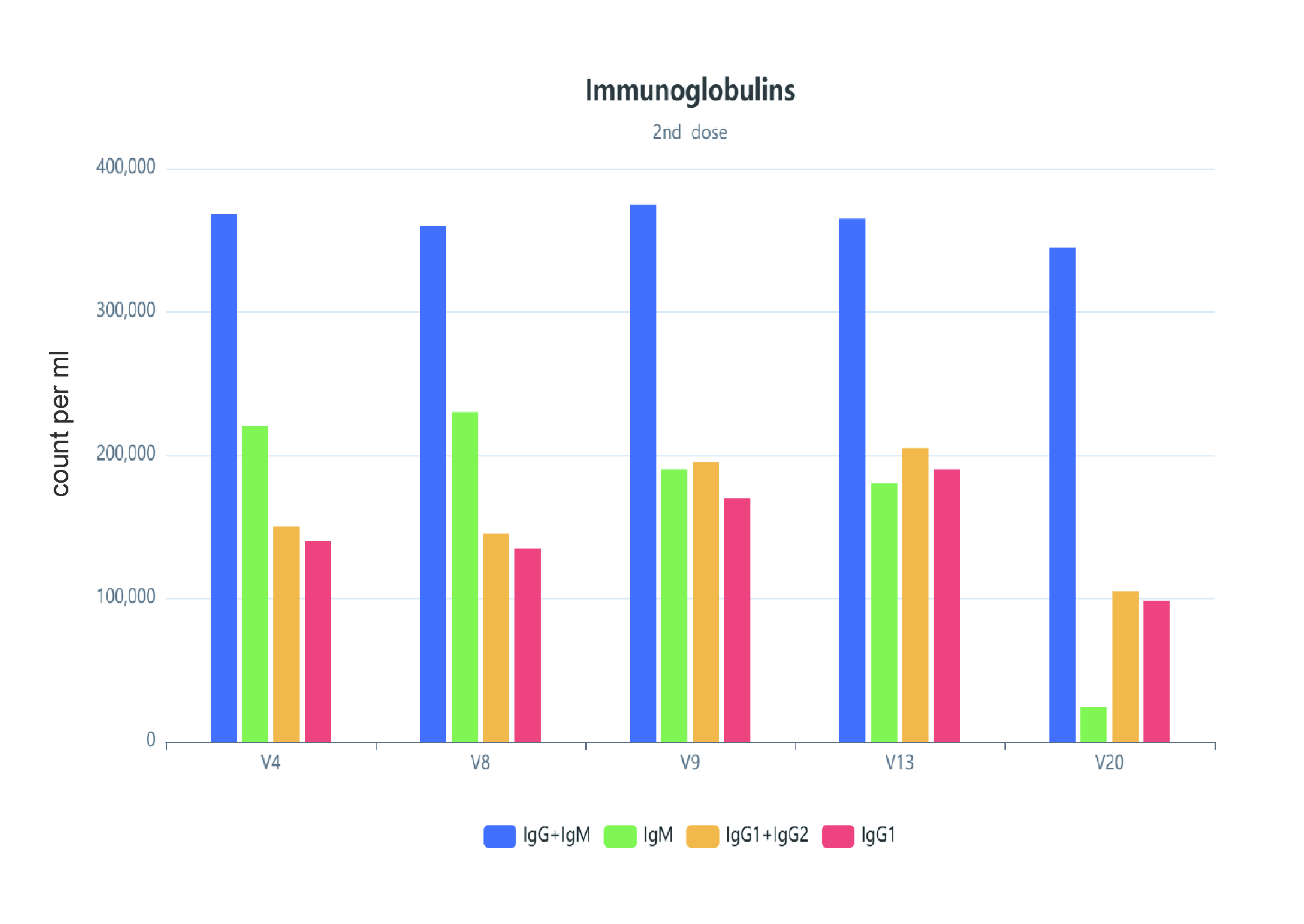
**

**
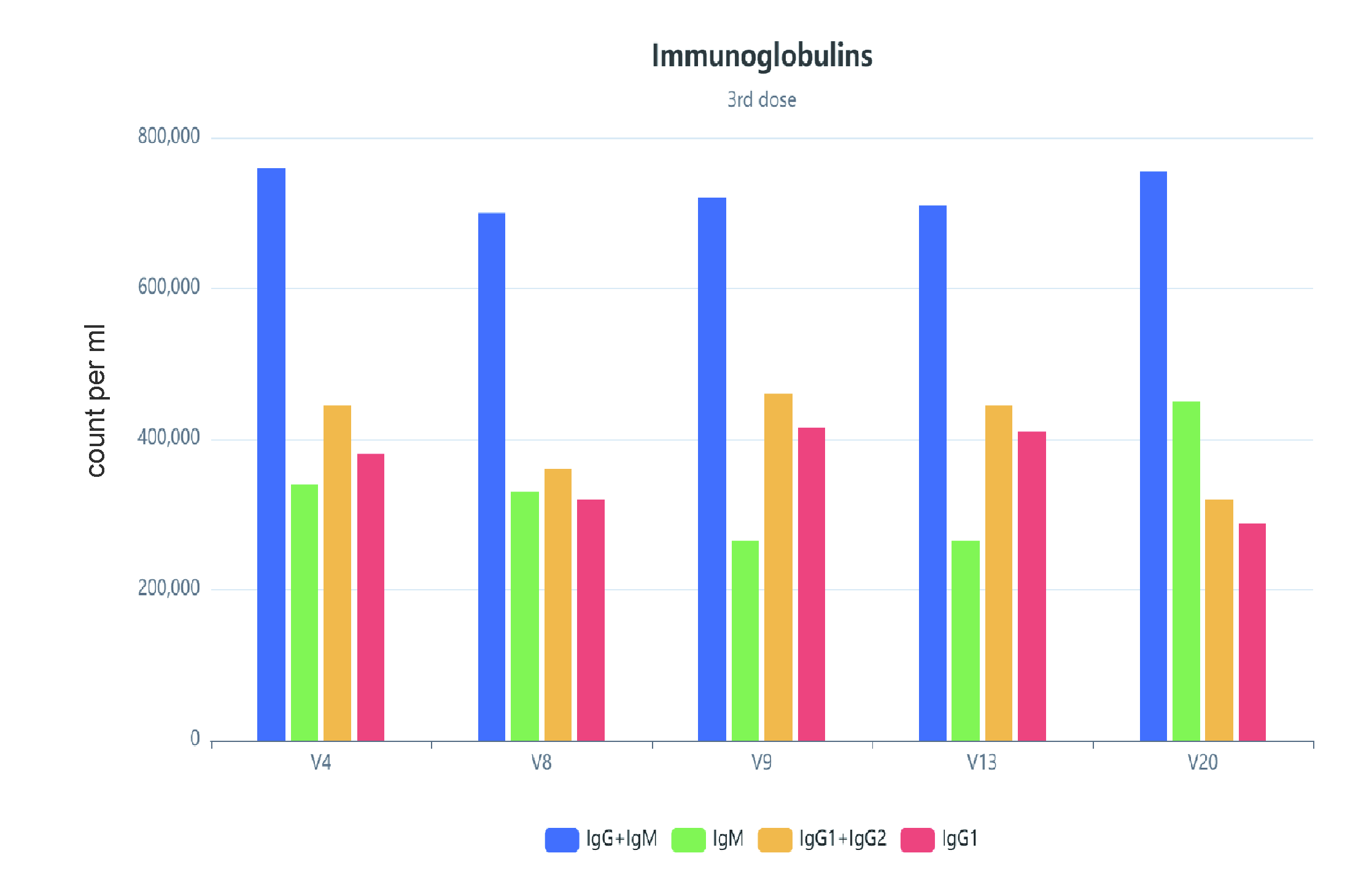
**

**
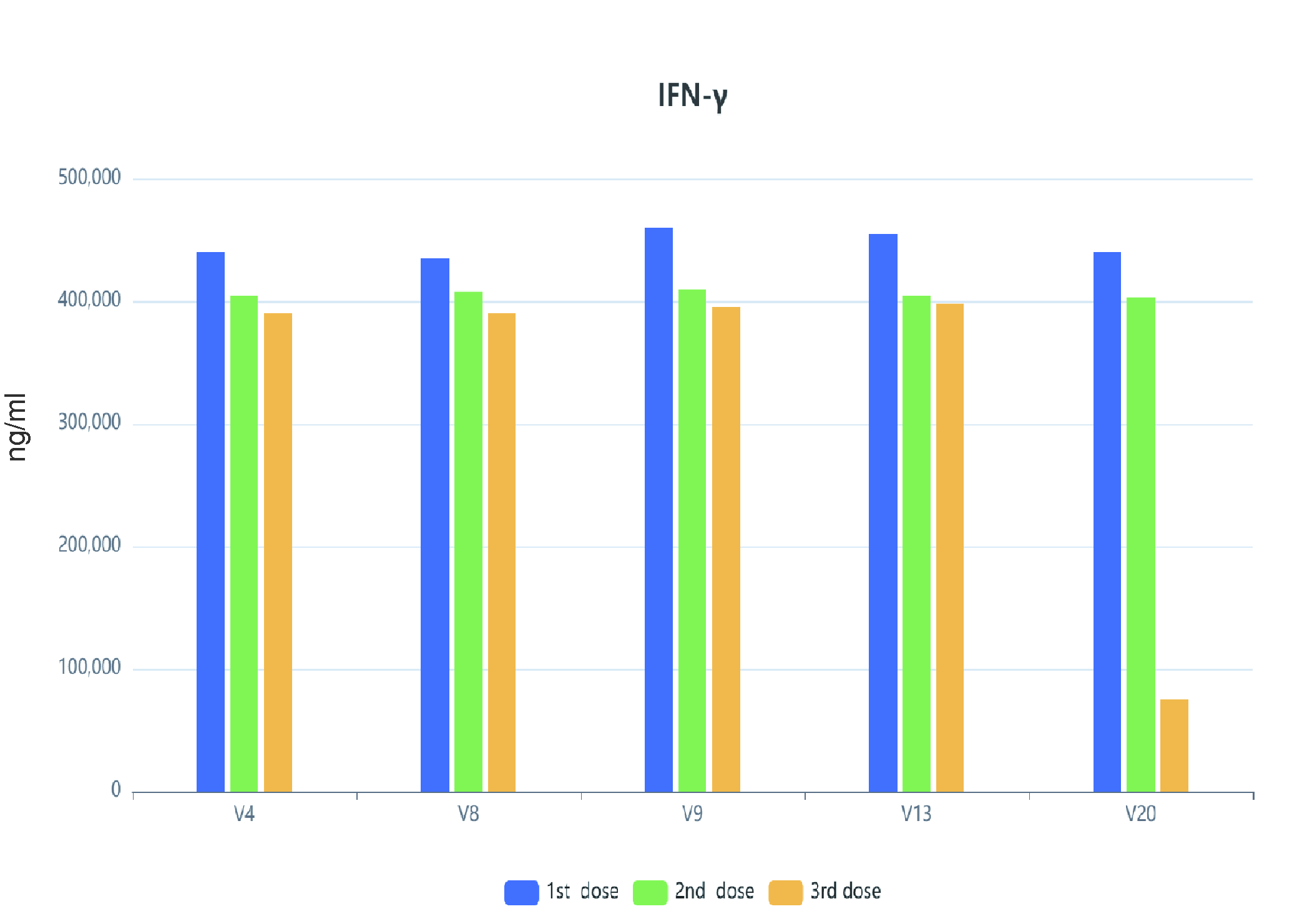
**

**From the above analysis, it is evident that the properties of the vaccine we constructed remained largely unchanged following the alteration in epitope ranking.**
